# Supplementary material for: Total delay and associated factors among tuberculosis patients in Jimma Zone, Southwest Ethiopia
Source: PLoS One. 2023 Feb 9;18(2):e0281546. doi: 10.1371/journal.pone.0281546 (PMC10045582; doi:10.1371/journal.pone.0281546)
Supplement: S1 Text — (PDF) [file pone.0281546.s001.pdf]

I. Request for participation in a research project for tuberculosis patient

"Performance and Quality of Tuberculosis Directly Observed Treatment Short Course (DOTS) Strategy in Jimma Zone, Southwest Ethiopia"

Introduction

My name is ___________________________I am working at _____________________________ health facility. I am kindly inviting you, to participate in a research study because you have been diagnosed to have a disease called tuberculosis (TB). The details of the research plan are described in this document. It is important that you understand why the research is being done and what it will involve. Please take your time to read through and consider this information carefully before you decide to participate in the proposed study. Please, ask if anything is unclear or if you would like to get more information.

Background and purpose

Tuberculosis (TB) is an infectious disease, responsible for serious illness and death globally, affecting both sexes of all age groups. TB is a common problem in Ethiopia including Jimma Zone. It is important to control the disease with feasible, cost effective, and acceptable approaches. The research project intends to assess the overall performance and quality of tuberculosis directly observed treatment short course (TB DOTS) strategy in Jimma Zone, Ethiopia. The knowledge obtained from this study will help us to provide useful information for decision makers so that the program performance and quality can be improved and the community members get better services. To be able to do this, learning about your experience and your knowledge as being a TB patient under treatment is very important. You are selected for the study from randomly selected Woredas (Districts).

What does the study entail?

In this study I would like to ask you some questions about the diagnostic process and your daily visits to this treatment site. The interview will last for about 50 to 60 minutes and take place at a time/place convenient for you. In addition, we would like to follow your treatment outcome until you complete the treatment and obtain information related with tuberculosis treatment as well as your treatment outcome status from TB registration book. This study does not affect your treatment and you will get your usual treatment whether you have participated in the study or not.

Potential advantages and disadvantages

You may spend about 50 to 60 minutes in providing us information about your background and process of tuberculosis diagnosis & treatment which may consume your precious time. There may not be any direct benefit as a result of your participation in the study, however your honest and genuine response will contribute to generate information that can be used to improve performance and quality of services related to tuberculosis.

What will happen to information about you?

The data that are registered about you will only be used in accordance with the purpose of the study. All the data will be processed without name, ID number or other directly recognisable type of information. A code number that links you to your data will be used, and the information you provide us will therefore be confidential. All data will be kept in a locked cabinet and password protected computers. In addition, your information will only be used during report writing and not after completing the project.

Voluntary participation

Participation in the study is voluntary. You can withdraw your consent to participate in the study at any time and without stating any particular reason. This will not have any consequences for your further treatment. If you wish to participate, sign the declaration of consent on the final page. If you agree to participate at this time, you may later on withdraw your consent without your treatment being affected in any way. If you later on wish to withdraw your consent or have questions concerning the study, you may contact:

Berhane Megerssa Ereso
Mobile phone number +251917804469
Email address – berhanemegerssa2004@gmail.com
Jimma University, Ethiopia

Supervisors

1. Mette Sagbakken (PhD, Associate professor)

Email address mette.sagbakken@nakmi.no
Mobile phone number +4741576964

2. Solomon Yimer (PhD, Postdoc)

Email address yimsolo@yahoo.com
Mobile phone number +4747687670

Releasing material and data to other parties

If you agree to participate in the study, you also consent de-identified data being released to local and regional health offices in Ethiopia.

Right to access and right to delete your data

If you agree to participate in the study, you are entitled to have access to what information is registered about you. You are further entitled to correct any mistakes in the information we have registered. If you withdraw from the study, you are entitled to demand that the collected data are deleted, unless the data have already been incorporated in analyses or used in scientific publications.

Funding and the role of Strategic and Collaborative Capacity Development in Ethiopia and Africa (SACCADE) Project

The study will be funded by research grants from Strategic and Collaborative Capacity Development in an Ethiopian and African (SACCADE) project. The SACCADE project will cover expenses related with personal, materials/ supplies and transportation. It does not have any conflict of interest with any other project.

Information about the outcome of the study

You are fully entitled to receive information about the result and outcome of this study.

Consent for participation in the study

I am willing to participate in the study

(Signature of the study participant, date)

Proxy consent (when necessary).

(Signature of guardian, date)

I confirm that I have given information about the study.

(Signature of data collector, date)

1. Questionnaire for Tuberculoses patient

Name of hospital/health center/health post _________________
Date of data collection_______________
Unit TB number (Code number) _____________________________
Woreda registry number_________________________________
Permanent address of the patient: Zone____ Woreda ___ Kebele___House no._____Phone no.______
Name of data collector____
Signature____

I. **Socio-demographic characteristics of the respondent**
First, I would like to ask you a few questions about yourself
For interviewer: please tick the box for the selected answer/s number/s additionally, write short answer in the blank space.

S.No	Questions	Response category	Skip
1	Sex of respondent	1. Male ---------------☐<br>2. Female-------------☐	
2	How old are you?	---------------------	
3	What is your current marital status	1. Single ---------------☐<br>2. Married -------------☐<br>3. Divorced ----------- ☐<br>4. Widowed ------------☐<br>5. Not applicable--------☐	
4	What is the highest level of education you have completed?	-----------------------------	
5	What is your occupation?	1. Farmer -------------------☐<br>2. Merchant ----------------☐<br>3. Government employee -☐<br>4. Daily laborer ------------☐<br>5. Others, please specify------	

S.No	Questions	Response category	Skip
6	What is your ethnic group?	1. Oromo ----------------☐<br>2. Amhara----------- -- ☐<br>3.Tigre-------------------☐<br>4.Yem -------------------☐<br>5. Dawuro ------------- ☐<br>6. Other please specify ------------	
7	What is your religion	1. Orthodox Christian------ ☐<br>2. Muslim -------------------☐<br>3. Protestant -----------------☐<br>4. Catholic ------------------☐<br>5.Other(specify) ---------------	
8	Where do you currently live? Please describe the name of your residence area	------------------------------------	
9	How far do you live from the nearest health facility that provides you tuberculosis treatment?	In kilometers ------------- or<br>in hours/minutes ----------	
10	What is your means of transportation to visit the health facility for tuberculosis treatment?	1. On foot ----------------- ☐<br>2. By car/ Bajaj -----------☐<br>3. Using horse/mule ------☐<br>4. By bus -----------------☐<br>5. Others (specify) --------	
11	Did you incur any cost for your daily visits to the clinic?	1. Yes ---------------------☐<br>2. No ----------------------☐	If 2,go to 14

S.No	Question	Response category	Skip
12	If yes, for Q 11, please mention the reason for payment?	1. For transport -------------------☐<br>2. For laboratory services -------☐<br>3. For food & accommodation --☐<br>4. Other (specify)-------------	
13	Would you please tell me total amount of money you have paid in relation to this illness?	-------------------------------	
14	How many family members are living in your household?	------------------------------	
15	Would you please tell me the number of rooms in the house that you are currently live in?	-------------------------------	
16	What is/are your means of income? Please describe	---<br>----------------------------	
17	How much do you approximately earn per year?	In cash -----------------------<br>In kind ----------------------------------<br>---<br>---	

II. Questions for assessing diagnostic delay among TB patients

I would like to ask you some questions to understand your experiences with your illness

S.No	Questions	Response category	Skip
18	When did you first experience to have cough?	-------------------------	
19	Before you come to the current health facility, did you visit any medical provider to get treatment for the cough?	1. Yes -----------------------☐<br>2. No ------------------------☐	If 2, go to 21
20	If yes for Q 19, which of the following medical providers did you first visit when you first had cough?	1. Health post ------------- ☐<br>2. Health centre -------------☐<br>3. Hospital -------------------☐<br>4. Private clinic ------------☐<br>5. Other, please describe --------	
21	Have you ever been in contact with a patient who has been taking drugs for treatment of tuberculosis?	1.Yes -------------------☐<br>2. No -------------------☐<br>3. I do not know/ I am not sure ☐	

22. Which of the following symptoms are you have been suffering from?

No	Symptoms	Yes	No	Duration of symptoms	Remark
22. 1	Dry cough				
22.2	Cough with whitish or yellowish sputum				
22.3	Cough with bloody sputum				
22.4	Chest pain				
22.5	Fever				
22.6	Night sweating				
22.7	Weight loss (10%)				
22.8	Loss of appetite				
22.9	Difficulty in breathing				
22.10	Other symptoms (specify)_______				

For interviewer, for questions 24 and 25 please cross check patient information with laboratory and TB registration books data

S.No	Questions	Response category	Skip
23	How long did it take from onset of the above symptoms until your first visit to a medical provider?	Days ---------------------<br>Weeks ----------------------<br>Months --------------------<br>Years ----------------------	
24	How long time passed between your first visit to a medical provider until first diagnosis of tuberculosis was made?	Hours -------------------<br>Days ---------------------<br>Weeks ----------------------<br>Months --------------------	
25	How long time passed between the first diagnoses of tuberculosis until you first started treatment?	Hours ---------- Days ----------------<br>Weeks ---------------Months -------	

S.No	Questions	Response category	Skip
26	**For the interviewer**: please fill the total time taken from patient's first start of TB symptoms until first start of treatment (Questions 23 + 24 + 25)	Days ---------------------<br>Weeks ----------------------<br>Months --------------------	
27	How do you describe your current illness status?	1. No limitation of day to day activity -------------- -----☐<br>2. Slight limitation of day to day activity ------------------- ☐<br>3. High limitation of day to day activity ------------☐	
28	Are you or have you ever been a smoker?	1. Yes ---------------------☐<br>2. No ---------------------☐	If 2, go to 30
29	If yes for Q 28, what type?	1. Shisha ------------------ ☐<br>(Duration ------------------)<br>2.Cigarette -----------------☐<br>(Duration -----------------)	
30	Do you drink alcohol at all?	1. Yes -------------------- ☐<br>2. No ----------------------☐	If 2, go to 32
31	If yes for Q 30, please mention the following:	Type of alcohol ----------------<br>Amount of alcohol consumed in millilitre per day ------------<br>Duration of alcohol consumption--	
32	Do you use or have you ever used khat?	1. Yes --------------- ☐<br>2. No -----------------☐	If 2, go to 34
33	If yes for Q 32, for how long?	-------------------------	
34	Have you evere lived or worked on a regular/temporary basis in a prison or camp?	1. Yes ---------------☐<br>2. No ----------------☐	If 2, go to 37
35	If "Yes" for Q 34, please specify	1. Prison -----------☐<br>2. Camp -----------☐	

S.No	Questions	Response category	Skip
36	If "Yes" for Q 34, for how long?	------------------------	
37	Have you ever been told by a doctor that you have diabetes mellitus?	1. Yes --------------- ☐<br>2. No -----------------☐	
38	Have you ever been told by a doctor that you have other chronic diseases?	1. Yes ------------------ ☐<br>2. No -------------------☐	If 2, go to Q 40
39	If yes for Q 38 , please specify	----------------------------	
40	Have you ever taken any previous anti-TB treatment?	1. Yes ------------------☐<br>2. No ------------------☐	
41	If yes for Q 40, how long did you take the treatment?	------------------------	

III. Tuberculosis knowledge and practice related questions

In the following, I would like to ask you some questions related to your opinion about tuberculosis disease and experience about TB care

S.No	Questions	Response category	Skip
42	In your opinion, who can be infected with TB?	1. Anybody --------------- ☐<br>2. Only poor people ------ ☐<br>3. Only people who consume much alcohol-----------------☐<br>4. Only people with HIV/AIDS -- ------------- -----------------☐<br>5. Only people who have been in prison ----------------------- ☐<br>6. Others, please explain --------	
43	What do you think are the signs and symptoms of tuberculosis? Please list	---<br>-------------------------------------	
44	Would you please tell me how a person can get tuberculosis?	-------------------------------------	
45	Is tuberculosis curable?	1.Yes ---------------------------☐<br>2. No -------------------------☐	

S.No	Questions	Response category	Skip
46	How can a person with TB be cured?	1. With herbal remedies -------☐<br>2. With pain killer ------------ ☐<br>3. With TB drugs through directly observed treatment short course☐<br>4. Do not know -------------------☐<br>5. Other (please explain) -------	
47	How long do you have to take TB treatment to become cured?	-----------------------------------	
48	What/who is/are your source of information about tuberculosis and its treatment?	1. Health care providers ---- ☐<br>2. TV/Radio ------------------☐<br>3. Family/relative ------------☐<br>4. Other (specify) -------------	
49	Have you ever faced any problem/ challenges in relation to the TB services provided for you?	1. Yes ----------------------☐<br>2. No -----------------------☐	If 2, go to 51
50	If yes to question No. 49, what is/are the problem/s you have faced yet?	1. Shortage of drugs -------☐<br>2. Absence of laboratory service - ---------------------------------☐<br>3. Unwanted effect of drugs -☐<br>4. Bad/impolite treatment by health personnel -------------- ☐<br>5. Poor information about the treatment ----------------------☐<br>6. Other (specify) ---------------	
**IV. Tuberculosis stigma related questions**<br>In the following, I would like to ask you some questions in relation to how you as a patient and other people in your community perceive TB			
51	Do you perceive yourself to be at risk of negative reactions due to your disease?	1. Yes ----------------------☐<br>2. No -----------------------☐	If 2, go to 53

S.No	Questions	Response category	Skip
52	If yes, for Q 51, from whom?	1. Myself --------------------☐<br>2. My family ----------------☐<br>3. Community ---------------☐<br>4. Health workers -----------☐<br>5. Other (please specify) ------	
53	Will you continue to take your anti-TB drugs in this health facility/post until completion of treatment?	1.Yes -------------------------☐<br>2. No -------------------------☐	If 1, go to 55
54	If "no" for Q 53, please indicate the name of the health facility that you will be taking your medications until the end of treatment	------------------------------------	

55. Being a tuberculosis patient, which of the following conditions or feelings have you felt/ experienced?

S. No	Feeling /experience	Yes	No	Explanation/justification
55.1	Considering myself as of less value			
55.2	Desire to keep others from knowing about my disease			
55.3	Hide my illness from my family			
55.4	Others think less of me			
55.5	Others have avoided me (Specify)			
55.6	I have isolated myself			
55.7	I have been asked to stay away from work			
55.8	I have lost job and/or reduced income			
55.9	Others have refused to visit me			
55.10	I think it may affect my marriage prospects			
55.11	I fear discrimination			
55.12	I have stayed away from work and/ or groups			
55.13	Other (specify)			

V. TB treatment and its outcome (to be filled by data collectors)

S.No	Questions	Response category	Skip
56	Type of DOTS	1. Facility based ----------☐<br>2. Community based --- ☐	
57	TB classification	1. Smear-positive pulmonary TB (PTB +ve) -☐<br>2. Smear-negative pulmonary TB (PTB–ve) -☐<br>3. Extra pulmonary TB (EPTB) ---------------☐	
58	TB diagnostic category	1. Bacteriological -----------------------☐<br>2. Histo-pathological ------------------☐<br>3. Radiological -------------------------☐<br>4. Other (specify)-------------	
59	TB treatment category	1. New --------------------------------☐<br>2. Relapse ----------------------------☐<br>3. Failure ------------------------------☐<br>4. Return after default ---------------☐<br>5. Other (specify) ---------------------	
60	Is there a contact person for follow-up registered with an address?	1. Yes ----------------------------☐<br>2. No -----------------------------☐	
61	HIV status of the patient	1. Reactive ------------------☐<br>2. Non-reactive --------------☐<br>3. Unknown ------------------☐	If 2or 3, go to 64
62	If Q No 61 is reactive, was ART stared before TB treatment?	1. Yes -----------------------☐<br>2. No ------------------------☐	

S.No	Questions	Response category	Skip
63	If yes for Q 62, when was ART started (please indicate the date of start)	______________________________________	
**Phase two (To be filled at the end of the treatment)**			
64	For smear-positive PTB patient, sputum result at end of 2nd or 3rd month	1. Negative -----------------☐<br>2. Positive -------------------☐<br>3. Not done -----------------☐	
65	For smear-positive PTB patient, sputum result at end of 5th month	1. Negative ------------ ------☐<br>2. Positive --------------------☐<br>3. Not done ------------------☐	
66	For smear-positive PTB patient, sputum result at end of the treatment	1. Negative ------------------☐<br>2. Positive -------------------☐<br>3. Not done ------------------☐	
67	Treatment outcomes	1. Cured ------------------------- ☐<br>2. Treatment completed --------☐<br>3. Died ----------------------------☐<br>4. Treatment failure------------☐<br>5. Default/lost to follow-up-----☐<br>6. Transferred-out ---------------☐<br>7. Other (specify) ---------------	

Date treatment started ____/___/____ Date treatment completed ____/___/_____

Name of data collector ______________________Date______/_______/________

Name of data supervisor ______________________Date______/_______/________

Thank you so much for your participation!

1. Gaaffii hirmaannaa qorannoo pirojektii Dhukkubsattoota "Tiibii' tiif dhiyaate

Raawwannaa fi qulqullina Tarsiimoo Koorsii gabaabaa yaaliinsa daawwannaa kallaattii dhukkuba "Tiibii'' (DOTS) Godina Jimmaa, kibba lixa itiyoophiyaa

Seensa

Maqaan koo---------------- -------------kani hojjadhu dhaabbatu fayyaa --------------------------------

Sababa dhukkuba 'Tiibii' jedhamee beekamu isin irratti waan argameefi hirmaannaa qo'annaa qorannoo irraatti akka hirmaattan kabajaan isin affeerreera. Tarreen karoora qorannoo kanaa ragaa kana irraatti tarreeffameera.Qorannoon kun maaliifi akka gaggeeffamu,maal of keessaa akka qabu, baruun isiniif barbaachisaa dha. Wixinee qorannoo irraatti hirmaachuuf murteessuu keessaaniin dura odeeffannoo kana of eeggannoon hubachuu fi waliigala isaa dubbisuuf adaraa yeroo kennaa! Wanti ifa isiniif hin taane yoo jiraate ykn Odeeffannoo dabalataa argachuuf gaafa dhaa.

Faayidaafi duub jalee

Dhikkubni "Tiibii dhukkuba daddarboo dha,akka waliigalaatti umrii kamiyyuu irraatti saala lamaanuu du'aafis ta'e dhukkuba cimaafi kan nama saaxilu dha.Dhukkubni "Tiibii'' godina jimmaa dabalatee itiyoophiyaa keessaatti beekamaa dha. Haala fudhatama,danda'amaafi baasii xiqqaa ta'een dhukkubicha to'achuun barbaachisaa dha. Yaadni piroojektii qorannoo kanaas;- itiyoophiyaa keessaa godina jimmaatti raawwii fi qulqullina waliigalaa tarsiimoo koorsii gabaabaa yaalinsa daawwannaa kallattii/'Tiibii'-DOTS/ sakattaa'uu dha. Miseensotni hawaasaa tajaajila gaarii akka argataniifi raawwiin fi qulqullina sagantichaa foyyeessuufi jecha qorannaa kana irraa beekumsi argamu odeeffannoo faayidaa qabeessa ta'e akka murtii kennitootaafi kenninu kan nu gargaaru dha. Kana hojjachuu akka dandeenyuufi waa'ee dhukkubsattoota 'Tiibii' yaalinsa irra jiranii muuxxaannoofi beekumsa keessaan argachuun baayyee barbaachisaa dha. Aanaalee tasaan filataman keessaa isin qorannoo kanaafi filatamtaniittu.

Qorannochi Maal qabata?

Qorannoo kana keessaatti ani gaaffiilee muraasa waa'ee adeemsa argannaa dhukkubichaafi kallaattii daawwannaa yaalinsaa guyyaa guyaanii keessan isin gaafadha. Gaaffifi deebiichi daqiiqaa 50 hanga 60 kan fudhatu yoo ta'u yeroofi bakkicha isiniif mijaa'aa ta'etti kan ta'u dha. Dabalataanis bu'aa yaalinsa keessanii hanga yaalisicha xumurtanitti ni hordofna, odeeffaannoo yaalinsa 'Tiibii'wojjiniin walqabate fi sadarkaa bu'ichaa galmee galmeessa 'Tiibii'irraa ni arganna. Qorannoo kana irraatti hirmaattanis dhiistanis yaalinsa argattan irraatti dhibbaa kamuu hin qabu.Yaalinsa Kanaan dura argattan itti fufa.

Jiraachuu Faayidaa fi miidhaa

Odeeffaannoo waa'ee eenyummaa keessanniifi adeemsa argannoofi yaalinsa 'Tiibii'nuuf kennitan yeroo keessan irraa daqiiqaa 50 hanga 60 ta'uu isin duraa gubachuu danda'a. Qorannoo kana irraatti hirmaachuu keessaaniifi kallattidhaan faayidaan isin argattan hin jiru ta'a garuu amanamummaafi bilisaan deebiin kennitan foyya'insa raawwii fi qulqullina tajaajila 'Tiibii'wojin walqabateef odeeffannoo gabbifachuufi gahee qaba.

Odeeffaannoo kennitan maal ta'uu danda'a?

Ragaan waa'ee keessan galmaa'ee kun faayidaa qorannoo kanaafi qofa oola. Ragaan kun kan raawwatu maqaafi lakkofsa eenyummaa kallaattiin haala odeeffaannoo hin beekamneeni dha. Lakkofsa addaa ragaa keessan isiniin wal qabsiisu ni fayyadamna,akkasumas odeeffaannoon isin nuuf kennitanu icitiin ni qabama,Ragaan hundu saanduqa keessatti furtuun kan qabamuufi lakkofsa icitii kompurataan ni eegama. Dabalataanis odeeffaannoon keessan kan fayyadamnu piroojektii eega xumurree booda osoo hin ta'in yeroo gabaasni barreeffamu qofa dha.

Hirmaannaa Fedhiinii

Hirmaanaan qorannoo kana keessaatti gaggeffamu fedhii irraatti kan hundaa'ee dha.Sababa dhuunfaa kamiyyuu osoo hin dhiheessin yeroo barbaaddan keessaatti fedhii keessan haquu ni dandeessu.Kunis yaalinsa itti fufu irraatti miidhaa wanta tokko isin irraatti hin qaqqabsiisu.hirmaachuu kan barbaaddan yoo ta'e fuula xumuraa waliigaltee irraatti mallateessaa.Yeroo kana keessaatti hirmaachuufi yoo waliigaltan, karaa kamiinuu yaalinsa keessan haala hin miineen booda irraatti waliigaltee keessan haquu ni dandeessu. Booda irraatti waliigaltee keessan haquu yoo barbaaddan ykn gaaffii qorannocha ilaallatu yoo qabaattan:

Birhaanee magarsaa Irreessoo

Lakkofsa mobaayilii 0917804469

Teessoo imeelii berhanemegerssa2004@gmail.com

Yunivarstii jimmaa, itiyoophiyaa

Supparvaayizaroota

1. Mette Sagbakken(PhD, pirofeesara asoosheetii)
   Teessoo imeelii mette.sagbkken@nakmi.no

   Lakkofsa mobaayilii +4741576964

2. Salamoon Yimar(PhD,postdoc)
   Teessoo imeelii yimsolo@yahoo.com
   Lakkofsa mobaayilii +4747687670

   Argachu ykn dubbisu dandeessu.

Miseensota kan birootiif ragaafi meeshaalee dabarsuu

Qorannoocha irraatti hirmaachuufi hanga waliigaltanitti ragaa eenyummaa hin ibsine itiyoophiyaatti w/ra fayyaa naannoofi godina keessaatti ragaa darbuufi eeyyamtanittu jechuu dha.

Mirga argachuufi haquu ragaa

Qorannocha irraatti hirmaachuuf hanga waliigaltanitti waa'ee keessanii odeeffannoo galmaa'ee argachuu ni dandeessu. Odeeffaannoo nuti galmeessine keessaatti dogongora kamiyyuu dabalataan sirreessuu ni dandeessu.Qorannocha keessaa bahuu yoo barbaaddan; hanga ragichi hin qindoofnetti ykn tajaajila maxxansaafi hin galleetti ragaa sassaabame haquu ni dandeessu.

Maallaqaafi gahee pirojektii tarsiimoo walgargaarsaa misooma dandeetti itiyoophiyaafi afriikaa (SACCADE)

Qo'annochi maallaqaan kan gargaaramu pirojektii tarsiimoo walgargaarsaa misooma dandeetti itiyoophiyaafi afriikaa (SACCADE) irraa qorannoofi kan argameetiini dha.Pirojektiin SACCADE baasiiwwan geejjibaa,namaafi dhiheessa meeshaaleetiin walqabatu ni aguuga.pirojektichi pirojektii kamiyyuu waliin walitti bu'iinsa faayidaa hin qabu.

Odeeffaannoo waa'ee bu'aa qo'annoochaa

Waa'ee xumuraafi bu'aa qo'annochaa odeeffaannoo fudhachuuf mirga guutuu qabdu.

Qo'annocha irraatti hirmaachuufi eeyyamaa ta'u

Ani qo'annocha irraatti hirmaachuufi eeyyameera.

(Mallaattoo hirmaataa qo'annichaa fi guyyaa)

Mallaattoo guddisaa fi guyyaa(yeroo barbaachisaa ta'e)

Waa'ee qo'annochaa odeeffaannoo kennuu koo nan mirkaneessa.

Mallaattoo sassaabaa Ragaa fi guyyaa

1. Gaaffii Dhukkubsataa 'Tiibiitiif'

Maqaa hospitaalaa/ buufata fayyaa/keellaa fayyaa________________________

Guyyaa sassaabbii raga__________________

Lakkoofsa yuniitii "Tiibii" /lakkoofsa koodii/ (unit TB No)_______________________________

Lakkoofsa galmee Aanaa, ____

Teessoo dhaabbataa dhukkubsataa: Godina ____________ Aanaa ________ Ganda ________ Lakk.Manaa ________ Lakk.Bilbilaa____________________

Maqaa raga sassaabaa ______________________mallaattoo ---------------------

I. Amaloota deebii kennaa walitti dhufeenya waa'ee ummataa

Duraan dursee, waa'ee keessan gaaffii muraasa isin gaafachuu nan barbaada.

Gaafataaf: Maloo sanduqaa kessaati deebii kessan mallaattoon agarsiisaa akkasumas, bakka duwwaa irraatti deebii gabaabaa kennaa.

**Lakk.s**	**Gaaffilee**	**Tarree deebii**	**Irraa darbu**
1	Saala deebii kennaa	1.Dhiira---------------☐<br>2.Dubara---------------☐	
2	Umriin keessan meeqa?	---------------------------------------	
3	Haalli gaa'ila keessanii yeroo ammaa maali?	1.Hin fuune/heerumne----------------☐<br>2.Fuudheera/heerumteetti-------------☐<br>3.Hiikeera/hikteetti---------------------☐<br>4.kan jalaa du'e/duute -----------------☐	
4	Sadarka barnoota isa ol'aanaa isin xumurtan kami?	______________________________________	
5	Hojiin keessan maali?	1.Qotee bulaa--------------------------☐<br>2. Daldalaa-----------------------------☐<br>3.Hojjata mootummaa----------------☐<br>4. Hojjataa guyyaa---------------------☐<br>5. kan biroo, (addaa baasaa) ---------------------------	

Lakk.s	Gaaffilee	Tarree deebii	Irraa darbu
6	Sabin keessan maali	1.Oromoo--------------------------------□<br>2.Amaaraa--------------------------------□<br>3.Tigiree---------------------------------□<br>4.Yeem-----------------------------------□<br>5.Dawuroo--------------------------------□<br>6.Kan biroo maaloo adda baasaa------□	
7	Amantiin keessan maali?	1.Kiristaana Orthoodooksii-------------□<br>2.Musiliima-------------------------------□<br>3.Pirooteestaantii-------------------------□<br>4.Kaatoolikii------------------------------□<br>5. Kan biro, maaloo adda baasaa-----□	
8	Yeroo ammaa eessa jiraattu? maaloo maqaa iddoo jiraattnii ibsaa.	---------------------------------	
9	Dhaabbatni fayyaa yaalinsa Tiibii isiniifi keennu irraa iddoon jiraattan hangam fagaata?	Kiloomeetiraan----------------------------<br>Sa`atiidhaan/daqiiqaa dhaan ------------	
10	Yaalinsa Tiibiitiifi dhaabbata Fayyaa yeroo dhaqxanu maaliin deemtu?	1.Miilaan----------------------------------□<br>2.Konkolaataa/bajaajii-------------------□<br>3.Fardaan/gaangeen----------------------□<br>4.Awwutoobisii ---------------------------□<br>5.kan biroo, adda baasi-------------------□	
11	Kilinika guyyaa guyyaan deemuuf baasiin isin baastanu jiraa?	1.Eeyyee-----------------------------------□<br>2.Lakkii------------------------------------□	Yoo 2 ta'e, gara 14
12	Gaaffii 11 f "eeyyee" yoo ta'e, sababa kaffaltii ibsa maaloo?	1.Geejjibaafi------------------------------□<br>2.Tajaajila laaboraatoaarfi--------------□<br>3.Nyaataa fi ciisichaaf------------------□<br>4. Kan biroo ibsii maaloo---------------□	

Lakk.s	Gaaffilee	Kutaa Deebii	Irraa darbu
13	Dhukkuba kanaan walqabatee hanga qarshii waliigalaa kaffaltan natti himuu dandeessuu?	--	
14	Miseensa maatii meeqatu mana keessan keessa jiraata?	--	
15	Mana amma keessa jiraattan keessa kutaalee meeqa akka qabu natti himuu dandeessuu?	---	
16	Haalli galii keessanii maali/maalfaa dha maaloo ibsaa?	--	
17.	Waggaatti tilmaamaan hangam argatta?	Qarshii caallan--------------------------------<br>Gosaan --------------------------------------	

II. Gaaffilee sakattaa'iinsaa dhukkubsattota Tiibii jidduu boodeessuu argama dhukkubichaa Dhukkubbii irraatti muuxxaannoo keessan hubachuufi gaaffilee muraasa isin gaafachuu barbaadna.

Lakk.s	Gaaffilee	Kutaa Deebii	Irraa darbu
18	Qufaan yeroo duraaf yoom isin jalqabee?	-------------------------------------	
19	Dhaabbata fayyaa kana dhufuu keessaniin dura yaalinsa qufaa argachuuf wal'aansaa kennaa kamiyyuu bira deemtanii beektuu?	1.Eeyyee ---------------------☐<br>2.Lakki-----------------------☐	Yoo 2 ta'e, gara 21
20	Yoo gaaffiin 19 eeyyee ta`e yeroo dura isin qufaasisu wal'aansa kennaa isa kam bira dhaqxan?	1. Keella fayyaa ----------------☐<br>2. Buufata fayyaa ---------------☐<br>3.hospitaala---------------------☐<br>4. kilinika dhuunfaa------------☐<br>5. kan biroo,maaloo ibsaa-----☐	

Lakk.s	Gaaffilee	Kutaa Deebii	Irraa darbu
21	Dhukkubsataa qoricha “Tiibii” fudhachaa ture waliin walitti dhufeenyaa qabdu tureeyii?	1.Eeyyee---------------------------☐<br>2 Lakki ---------------------------☐<br>3. Hin beeku /sirritti hin beeku/-☐	

22. Mallattoolee armaan gadii keessaa hanga ammatti isa kamtu isin rakkisaa jira?

Lakk	Mallattoolee	Eeyyee	Lakki	Turtii mallattoolee	Yaada
22.1	Qufaa gogaa				
22.2	Qufaa hakkee adii yookiin keelloo qabu				
22.3	Qufaa hakkee dhiiga qabu				
22.4	Waraansa laphee				
22.5	Ho`ina qaama				
22.6	Halkaan dafqisiisuu				
22.7	Hir`ina ulfaatina (10/%)				
22.8	Fedhii nyaataa dhabuu				
22.9	Rakkoo afuura baafachuu				
22.10	Mallaattoo kan biroo/ibssa/----------				

Gaaffii dhiyeessaaf, Gaaffiilee 24 fi 25 maaloo odeeffannoo dhukkubsaticha isa laabiraatoorii fi galmeessa ragaa ‘Tiibii’ waliin mirkaneessa.

Lak k.s	Gaaffilee	Kutaa deebii	Irraa darbu
23	Mallattoo dhukkuba armaan olii eega isin mudate yeroo hangamitti waldhaansa kennitootaa bira dhaqxan?	Guyyoota --------------------<br>Torbanoota -----------------<br>Ji’oota ----------------------<br>Waggoota------------------	
24	Yeroo duraafi waldhaansaa ‘Tiibii’ kennitoota bira dhaquufi argamuu ‘Tiibii’ jidduu yeroo hangam ture?	Sa’atii--------------------<br>Guyyoota----------------<br>Torbanoota--------------<br>Ji’oota ------------------	
25	Yeroo duraa fi mu’lachuu ‘Tiibii’ fi jalqabuu yaalinsa jidduu yeroo hangamtu ture?	Sa’atii-----------------<br>Guyyoota--------------<br>Torbanoota--------------<br>Ji’oota -----------------	

Lak k.s	Gaaffilee	Kutaa Deebii	Irraa darbu
26	**Gaaffii dhiyeessaaf :** Maaloo jalqabuu mallaattoo 'Tiibii'fi jalqabuu yaalinsaa 'Tiibii' jidduu yeroo hangam akka fudhate guutaa (gaaffii 23 + 24 + 25)	Torbanoota ----------------<br>Guyyoota------------------<br>Ji'oota ---------------------	
27	Haala/sadarkaa/ dhukkuba keesan yeroo ammaa akkamiitti ibsitu?	1.Sochii guyyaa guyyaatiin garaagarummaa hin qabu-----------------------------------☐<br>2.Sochii guyyaa guyyaatiin hanga tokko ana daangeesseera.-------------------- ☐<br>3. Sochii guyyaa guyyaatiin bayyee ana daangeesseera ----------------☐	
28	Ni xuuxxuu turee ykn xuuxaa jirtuu?	1.Eyyee ---------------------☐<br>2.Lakki ----------------------☐	Yoo 2 ta'e, gara 30
29	Gaaffii 28 ffafi "eeyyee": yoo ta'e , gosa kami?	1. Shiishaa -----------------☐ (turtii) ---------<br>2.Tamboo ----------------☐ (turtii) ---------	
30	Dhugaatii alkoolii ni dhugduu?	1.Eyyee -------------------☐<br>2.Lakki ------------------☐	Yoo 2 ta'e, gara 32
31	Yoo gaaffiin 30ffaa "eeyyee" yoo ta`e, maaloo kan itiannu ibssa?	Gosa alkoolii -------------------<br>Hanga alkoolii guyyatti mililitraan fudhattan ---<br>Yeroo hangamiif fudhattan ---------------	
32	Caatii ni fayyadamtuu ykn fayyadamtanii beektuu?	1.Eeyyee -------------------☐<br>2.Lakkii--------------------☐	Yoo 2 ta'e, gara 34
33	Yoo gaaffiin 32 eeyyee ta'e, yeroo hagamiif?	---------------	
34	Mana sirreessa ykn kaampii dhaabbataan/yeroof jiraattanittuu ykn hojjattanittuu?	1.Eyyee --------------------☐<br>2.lakkii ---------------------☐	Yoo 2 ta'e, gara

			37
**Lak k.s**	**Gaaffilee**	**Kutaa Deebii**	**Irraa darbu**
35	Yoo gaaffiin 34ffaa “eeyyee” ta’e Maaloo ibsaa---	1.Mana sirreessa----------☐<br>2. Kaampii -----------------☐	
36	Yoo gaaffiin 34 “eeyyee” ta’e, yeroo hangamiif?	----------------------	
37	Akka dhukkuba sukkaaraa isin keessa jiru haakimiin isinitti himee beekaa?	1. Eeyyee------------------☐<br>2. Lakki -------------------☐	
38	Akka dhukkuboota nama irra turanii isin keessa jiru haakimiin isinitti himee beekaa?	1. Eeyyee--------------------☐<br>2. Lakki ---------------------☐	Yoo 2 ta’e, gara 40
39	Gaaffii 38 “eeyyee” yoo ta’e maaloo ibsaa ------------	----------------------	
40	Yaaliinsa qoricha ‘Tiibii’ kamuu kanan dura fudhattanii beektuu?	1.Eeyyee---------------------☐<br>2. Lakkii.---------------------☐	Yoo 2 ta’e, gara 42
41	Gaaffii 40ffaaf “eeyyee” yoo ta’e yaalinsicha yeroo hangamiif fudhattan?	-------------------------	

III. Gaaffilee Beekumsaa fi gochaa ‘Tiibii’ waliin walqabtee

Waa’ee kunuunsa ‘Tiibii’muuxxaannoo qabdanu fi waa’ee dhukkuba ‘Tiibii’ Yaada qabdanu gaaffii muraasa haala armaan gadiin isin gaafachuu barbaanna.

**Lakk.s**	**Gaaffilee**	**Kutaa Deebii**	**Irraa darbu**
42	Akka yaada keessaniitti eenyutu dhukkuba ‘Tiibii’dhaan qabamuu danda’a?	1. Nama kamiyyuu--------------------------------------☐<br>2.Hiyyeessoota qofa-------------------------------------☐<br>3.Nama dhugaatii alkooli baayyee fudhatu qofa----☐<br>4. Nama HIV /AIDS qabu qofa------------------------☐<br>5.Nama mana sirreessa ture qofa----------------------☐<br>6. ka biroo, ibsa ---------------------------------------	
43	Mallattoolee dhukkuba‘Tibii’ maalifaa dha jeetani yaaddu?	---<br>--<br>---	

**Lakk.s**	**Gaaffilee**	**Kutaa Deebii**	**Irraa darbu**
44	Namni Tiibii'n qabamuu akkamitti akka danda`u natti himu ni dandeessuu?	---	
45	'Tiibii'n kan fayyudhaa?	1.Eeyyee --☐<br>2.Lakki---☐	
46	Namni Tibii qabu akkamiin fayyuu danda`aa?	1.Wal'aansa naannootiin /herbal remedies/ --------☐<br>2. Dhukkubbii tasgabbeessatiin ----------------------☐<br>3.Qoricha 'Tiibii'koorsii gabaabaa yaalinsa hordoffii kallaattiin kennamuutiin ---------------------------- ☐<br>4. Hin beeku ---☐<br>5. Kan biroo /maaloo ibsa ----------------------------	
47	'Tiibii' irraa fayyuufi yeroo hangamiifi yaalinsicha fudhachuu qabdu?	___________________________________	
48	Maddi odeeffannoo waa'ee Tiibiifi yaalinsa isaa eenyuu irraa/maalfaa dha?	1. Kennitoota kunuunsa fayyaa -------------------☐<br>2. TV/Reediyoo-------------------------------------☐<br>3. Maatii/fira--☐<br>4. Kan biroo/ibsaa ----------------------------	
49	Tajaajiloota'Tibii' wojjiniin walqabatan yeroo isinii kennamu rakkoon isin qunnamee beekaa?	1.Eeyyee -- ☐<br>2.Lakkii--☐	Yoo 2 ta'e, gara 51
50	Yoo gaaffii 49ffaaf "eeyyee" ta'e rakkoo/lee hanga yoonaa isn qunname maalidha/maalfadha?	1. Hanqina qorichootaa ---------------------------------☐<br>2. Dhabiinsa tajaajila laabiraatoorii -------------------☐<br>3. Bu'aa qorichaa kan hin barbaadamne--------------☐<br>4.Yaalinsa amala badaa ogeessa fayyaatiin ----------☐<br>5. Waa`ee yaalinsaa odeeffaannoon dadhabaa ta'uu ☐<br>6. Kan biroo/ibsi-------------------------------	

IV. Gaaffilee dhiibbaa hawaasa 'Tiibii' waliin walqabate

Haala armaan gadiitiin isin akka dhukkubsataa tokkoo fi namoota biroo hawaasa keessaatti 'Tiibii'akkmitti akka hubatanu isin gaafachuu nan barbaada.

**Lakk.s**	**Gaaffilee**	**Kutaa Deebii**	**Irraa darbu**
51	Dhukkuba keessan kana ilaalchisee dhibbaaf saaxilameera jettani of hubattani beektu?	1. Eeyyee -- ☐<br>2. Lakkii --- ☐	Yoo 2 ta'e, gara 53
52	Gaaffii 51ffafi eeyyee yoo ta'e gama kamiini dha?	1. Ofii kootiin ------------------------------------ ☐<br>2. Maatii koo irraa--------------------------------- ☐<br>3. Hawaasa irraa ---------------------------------- ☐<br>4. Hojjataa fayyaa irraa---------------------------- ☐<br>5. Kan biroo/maaloo ibsaa --------------------------	
53	Hanga yaalinsichi xumuramutti dhaabbata fayyaa/ kellaa fayyaa kanatti qoricha 'Tiibii'fudhachuu itti fuftuu?	1.Eeyyee -- ☐<br>2.Lakki --- ☐	Yoo 1 ta'e, gara 55
54	Gaaffii 59 f Yoo "Lakki" ta'e hanga yaalinsichi xumuramutti maqaa dhaabbata fayyaa qorcha itti fudhattan maaloo ibsa.	____________________________________	

55. Akkuma dhukkubsataa 'Tiibii' taatanitti haalawwan/mirawwan armaan gadii keessaa isa kamtu isnitti dhagahame/ isin qunname ture?

**Lakk.**	**mudannoo/wanta itti dhagahamu**	**Eeyyee**	**lakkii**	**Ibsama**
55.1.	Akka gadi aanaati ofi ilaaluu			
55.2.	Namootni biroo waa'ee dhukkuba koo akka hin beekne barbaadu			
55.3.	Dhukkuba koo maatii koo dhoksuu			

**Lakk.**	**mudannoo/wanta itti dhagahamu**	**Eeyyee**	**lakkii**	**Ibsama**
55.4.	Namootni biroo ana gadi qabani ilaaluu			
55.5	Namootni biroo anan fageesuu/ibsa			
55.6.	Ofii koo of addaan fo'uu			
55.7.	Hojiin ala turuufi gaafadheen ture			
55.8.	Hojii koo dhiiseera fi/ykn galiin koo xiqqaateera			
55.9.	Namootni biroo ana gaafachuu dhisaaniru			
55.10	Fudhatama ga'ila koo irraatti dhiibbaa uumeera natti fakkaataa?			
55.11	Qoqoodinsa nan sodaadha.			
55.12	Hojii koo irraa fi/ykn hiriyoota koo irraa fagaadheera.			
55.13	Kan biroo/ibsi			

V. Yaalinsa 'Tiibii' fi bu'aa isaa /sassaabaa ragaatiin kan guutamu/

**Lakk.s**	**Gaaffilee**	**Kutaa Deebii**	**Irraa darbu**
56	Gosa 'DOTS'	1. Dhaabbata kan bu'uureeffate ---------☐<br>2. Hawaasa kan bu'ureeffate ------------☐	
57	Kutaalee 'Tiibii'	1.'Tiibii' sombaa hakkee irraatti argame/PTB+ve -----------------------------☐<br>2.'Tiibii' sombaa hakkeen hin argamne (PTB-ve)-- ☐<br>3.'Tiibii'sombaa kan hin taane (EPTB)-- ☐	
58	Kutaalee argama dhukkuba 'Tiibii'	1. Baakteeriyaadhaan -----------------☐<br>2. Histoo-paatooloojiidhaan----------☐<br>3. Raajiidhaan/Raadiyooloojiidhaan/☐<br>4. Kan biroo, ibsaa ------------------	

59	Kutaalee yaaliinsa ‘Tiibii’	1. Haaraa --------------------------------□<br>2. Kan itti Deebi’ee/relapse/ ----------□<br>3. Milkaa’uu hafuu/failure/------------□<br>4. Addaan kutanii deebi’uu------------□<br>5. Kan biroo ibsaa ---------------------	
**Lakk.s**	**Gaaffilee**	**Kutaa Deebii**	**Irraa darbu**
60	Hordoffifi namni waamamuu danda’u teessoo waliin galmaa’ee jiraa?	1. Eeyyee -------------------------------□<br>2. Lakkii---------------------------------□	
61	HIV qabaachuuf dhabuu dhukkubsattoota	1. Qaba ---------------------------□<br>2. Hin qabu-----------------------□<br>3. hin beekamne------------------□	Yoo 2 ykn 3 ta’e, gara 64
62	Gaaffii 61ffaa yoo ‘qaba’ ta’e yaalinsi ‘Tiibii’ osoo hin jalqabmin dura qorichi isaa/ART/jalqabameeraa?	1. Eeyyee -----------------------□<br>2. Lakki -------------------------□	
63	Gaaffii 62ffafi yoo eeyyee ta’ee qorichi (ART) yoomi jalqabame(maaloo guyyaa jalqabamee ibsaa)	------------------------------	
**Marsaa lamaffaa (xumura yaalinsaa irraatti kan guutamu)**			
64	Dhukubsattoota Tiibii sombaa hakkeedhaan pozatiivii ta’an Ji’a xumura 2ffaa ykn 3faa irraatti bu’aan hakkee isaanii maali dha?	1. Nagatiivii-------------------------□<br>2. Pozatiivii-------------------------□<br>3. Hin hojjatamne------------------□	
65	Dhukubsattoota Tiibii sombaa hakkeedhaan pozatiivii ta’an Ji’a xumura 5ffaa irraatti bu’aan hakkee isaanii maali dha?	1.Nagatiivii------------------------------□<br>2.Pozatiivii-------------------------------□<br>3.Hin hojjatamne------------------------□	
66	Dhukubsattoota Tiibii sombaa hakkeedhaan pozatiivii ta’an yaalinsa xumura irraatti bu’aan hakkee isaanii maali dha?	1.Nagatiivii------------------------------□<br>2.Pozatiivii------------------------------□	

		3.Hin hojjatamne----------------------- □	
67	Bu’aa yaalinsaa	1. Fayyeera. ------------------------ □<br>2. Yaalinsi xumurameera.--------- □<br>3. Du’eera. -------------------------- □<br>4. Yaalinsi hin milkoofne.------- □<br>5. Hordoffiin addaan citeera.----- □<br>6. Gadhiiseera/transferred out/---- □<br>7. Kan biroo/ibsi/----------------------	

Guyyaa yaalinsi jalqabe -----------/------/------- guyyaa yaalinsi xumurame -------/-----------/---------

Maqaa ragaa sassaabaa -------------------------------------guyyaa --------/------------/----------------------

Maqaa supparvaayizarii ragaa --------------------------guyyaa ---------/-------------/------------------

Hirmaannaa keessaaniif baayyee isin galateeffadha!
