# Supplementary material for: Total delay and associated factors among tuberculosis patients in Jimma Zone, Southwest Ethiopia
Source: PLoS One. 2023 Feb 9;18(2):e0281546. doi: 10.1371/journal.pone.0281546 (PMC10045582; doi:10.1371/journal.pone.0281546)
Supplement: Attachment — Submitted filename: PONE-D-22-02817-commented.pdf [file pone.0281546.s006.pdf]

PLOS ONE

To[illegible] delay and associated factors among tuberculosis patients in Jimma Zone, Southwest Ethiopia

--Manuscript Draft--

**Manuscript Number:**	PONE-D-22-02817
**Article Type:**	Research Article
**Full Title:**	Total delay and associated factors among tuberculosis patients in Jimma Zone, Southwest Ethiopia
**Short Title:**	Total delay and associated factors among tuberculosis patients
**Corresponding Author:**	Berhane Megerssa Ereso, MSc, PhD candidate<br>Jimma University<br>Jimma, ETHIOPIA
**Keywords:**	Tuberculosis, total delay, diagnostic delay, treatment delay, Jimma Zone, Ethiopia
**Abstract:**	Background: Delays in diagnosis and treatment of tuberculosis (TB) increases severity of illness and continued transmission of TB in the community. Understanding the magnitude and factors associated with total delay is imperative to expedite case detection and treatment of TB.<br>Methods: Analytic cross-sectional study was conducted in Jimma Zone, Southwest Ethiopia. All newly diagnosed TB patients > 15 years of age were prospectively enrolled from randomly selected eight districts and one town in the study area. A structured questionnaire was applied to collect socio-demographic and clinical data. The median total delay was used to dichotomize the sample into delayed and non-delayed patient categories. Logistic regression analysis was used to analyse the association between independent and outcome variables. A p-value < 0.05 were considered statistically significant.<br>Results: a total of 1,161 patients were enrolled in this study. The median total delay was 35 days. Patients who had swelling or wound in the neck region were more likely to be delayed than their counterpart [adjusted odds ratio (AOR) =3.02, 95% confidence interval (CI): 1.62, 5.62]. Women were more likely to experience longer total delay (AOR =1.46, 95% CI:1.00,2.14) compared to men. Patients who had poor knowledge of TB were more likely to be delayed compared to those who had good knowledge (AOR=3.92, 95% CI: 2.65, 5.80).<br>Conclusion: the study showed unacceptably long total delay. Targeted interventions that enhance TB knowledge and practice, expedite early suspect identification, referral and management of all forms of TB is imperative to reduce total delay in diagnosis and treatment of TB.
**Order of Authors:**	Berhane Megerssa Ereso, MSc, PhD candidate
	Mette Sagbakken, PhD
	Christoph Gradmann, PhD
	Solomon Abebe Yimer, PhD
**Additional Information:**	
**Question**	**Response**
**Financial Disclosure**<br><br>Enter a financial disclosure statement that describes the sources of funding for the work included in this submission. Review the submission guidelines for detailed requirements. View published research articles from *PLOS ONE* for specific examples.	This study was not funded by a grant. It is a PhD project and was supported by the Strategic and Collaborative Capacity Development in Ethiopia and Africa (SACCADE) Project, Norwegian Program for Capacity Development in Higher Education and Research for Development (NORHAD), University of Oslo.

Powered by Editorial Manager® and ProduXion Manager® from Aries Systems Corporation

This statement is required for submission and **will appear in the published article** if the submission is accepted. Please make sure it is accurate.<br><br>**Unfunded studies**<br>Enter: *The author(s) received no specific funding for this work.*<br><br>**Funded studies**<br>Enter a statement with the following details:<br>• Initials of the authors who received each award<br>• Grant numbers awarded to each author<br>• The full name of each funder<br>• URL of each funder website<br>• Did the sponsors or funders play any role in the study design, data collection and analysis, decision to publish, or preparation of the manuscript?<br>• **NO** - Include this sentence at the end of your statement: *The funders had no role in study design, data collection and analysis, decision to publish, or preparation of the manuscript.*<br>• **YES** - Specify the role(s) played.<br><br>* typeset	
**Competing Interests**<br><br>Use the instructions below to enter a competing interest statement for this submission. On behalf of all authors, disclose any competing interests that could be perceived to bias this work—acknowledging all financial support and any other relevant financial or non-financial competing interests.<br><br>This statement is required for submission and **will appear in the published article** if the submission is accepted. Please make sure it is accurate and that any funding sources listed in your Funding Information later in the submission form are also declared in your Financial Disclosure statement.<br><br>View published research articles from	The authors have declared that no competing interests exist. There are no financial or non-financial competing interests among the authors.

PLOS ONE for specific examples.<br><br>**NO authors have competing interests**<br><br>Enter: *The authors have declared that no competing interests exist.*<br><br>**Authors with competing interests**<br><br>Enter competing interest details beginning with this statement:<br><br>*I have read the journal's policy and the authors of this manuscript have the following competing interests: [insert competing interests here]*<br><br>* typeset	
**Ethics Statement**<br><br>Enter an ethics statement for this submission. This statement is required if the study involved:<br><br>• Human participants<br>• Human specimens or tissue<br>• Vertebrate animals or cephalopods<br>• Vertebrate embryos or tissues<br>• Field research<br><br>Write "N/A" if the submission does not require an ethics statement.<br><br>General guidance is provided below. Consult the submission guidelines for detailed instructions. **Make sure that all information entered here is included in the Methods section of the manuscript.**	Ethical clearance was sought from the Regional Committee for Medical Research Ethics (REK Øst), Norway with reference number of 2015/2124 REK sør-øst B. Ethical approval was also obtained from the Institutional Review Board of Jimma University, Ethiopia with reference number of RPGC/389/2016. Written and oral informed consent was secured from each study participant before starting the data collection.

**Format for specific study types**<br><br>**Human Subject Research (involving human participants and/or tissue)**<br>• Give the name of the institutional review board or ethics committee that approved the study<br>• Include the approval number and/or a statement indicating approval of this research<br>• Indicate the form of consent obtained (written/oral) or the reason that consent was not obtained (e.g. the data were analyzed anonymously)<br><br>**Animal Research (involving vertebrate animals, embryos or tissues)**<br>• Provide the name of the Institutional Animal Care and Use Committee (IACUC) or other relevant ethics board that reviewed the study protocol, and indicate whether they approved this research or granted a formal waiver of ethical approval<br>• Include an approval number if one was obtained<br>• If the study involved *non-human primates*, add *additional details* about animal welfare and steps taken to ameliorate suffering<br>• If anesthesia, euthanasia, or any kind of animal sacrifice is part of the study, include briefly which substances and/or methods were applied<br><br>**Field Research**<br><br>Include the following details if this study involves the collection of plant, animal, or other materials from a natural setting:<br>• Field permit number<br>• Name of the institution or relevant body that granted permission	
**Data Availability**<br><br>Authors are required to make all data underlying the findings described fully available, without restriction, and from the time of publication. PLOS allows rare exceptions to address legal and ethical concerns. See the PLOS Data Policy and FAQ for detailed information.	Yes - all data are fully available without restriction

Powered by Editorial Manager® and ProduXion Manager® from Aries Systems Corporation

A Data Availability Statement describing where the data can be found is required at submission. Your answers to this question constitute the Data Availability Statement and **will be published in the article**, if accepted.<br><br>**Important:** Stating 'data available on request from the author' is not sufficient. If your data are only available upon request, select 'No' for the first question and explain your exceptional situation in the text box.<br><br>Do the authors confirm that all data underlying the findings described in their manuscript are fully available without restriction?	
**Describe where the data may be found in full sentences. If you are copying our sample text, replace any instances of XXX with the appropriate details.**<br><br>• If the data are **held or will be held in a public repository**, include URLs, accession numbers or DOIs. If this information will only be available after acceptance, indicate this by ticking the box below. For example: *All XXX files are available from the XXX database (accession number(s) XXX, XXX.)*.<br>• If the data are all contained **within the manuscript and/or Supporting Information files**, enter the following: *All relevant data are within the manuscript and its Supporting Information files.*<br>• If neither of these applies but you are able to provide **details of access elsewhere**, with or without limitations, please do so. For example:<br><br>*Data cannot be shared publicly because of [XXX]. Data are available from the XXX Institutional Data Access / Ethics Committee (contact via XXX) for researchers who meet the criteria for access to confidential data.*<br><br>*The data underlying the results presented in the study are available from (include the name of the third party*	All data analyzed during the current study are available from the corresponding author on request.

and contact information or URL).<br>• This text is appropriate if the data are owned by a third party and authors do not have permission to share the data.<br><br>* typeset	
Additional data availability information:	

Manuscript Click here to access/download;Manuscript;Manuscript.docx

Total delay and associated factors among tuberculosis patients in Jimma Zone, Southwest Ethiopia

Berhane Megerssa Ereso*[1, 2] ; Mette Sagbakken[3] ; Christoph Gradmann[1]; Solomon Abebe Yimer[4, 5]

1. @Department of Community Medicine and Global Health, Institute of Health and Society, University of Oslo, Oslo, Norway
2. #Department of Health Policy and Management, Faculty of Public Health, Institute of Health, Jimma University, Jimma, Ethiopia
3. @Department of Nursing and Health Promotion, Faculty of Health Sciences, Oslo Met- Oslo Metropolitan University, Oslo, Norway
4. @Department of Microbiology, Institute of Clinical Medicine, University of Oslo, Oslo, Norway
5. @ Coalition for Epidemic Preparedness Innovations (CEPI), Oslo, Norway

*Corresponding author

E- mail: berhanemegerssa2004@gmail.com (BME)

#: Current address of corresponding author, @: Current address for other authors

Abstract

***Background**: Delays in diagnosis and treatment of tuberculosis (TB) increases severity of illness and continued transmission of TB in the community. Understanding the magnitude and factors associated with total delay is imperative to expedite case detection and treatment of TB.*

***Methods**: Analytic cross-sectional study was conducted in Jimma Zone, Southwest Ethiopia. All newly diagnosed TB patients > 15 years of age were prospectively enrolled from randomly selected eight districts and one town in the study area. A structured questionnaire was applied to collect socio-demographic and clinical data. The median total delay was used to dichotomize the sample into delayed and non-delayed patient categories. Logistic regression analysis was used to analyse the association between independent and outcome variables. A p-value < 0.05 were considered statistically significant.*

***Results**: a total of 1,161 patients were enrolled in this study. The median total delay was 35 days. Patients who had swelling or wound in the neck region were more likely to be delayed than their counterpart [adjusted odds ratio (AOR) =3.02, 95% confidence interval (CI): 1.62, 5.62]. Women were more likely to experience longer total delay (AOR =1.46, 95% CI:1.00, 2.14) compared to men. Patients who had poor knowledge of TB were more likely to be delayed compared to those who had good knowledge (AOR=3.92, 95% CI: 2.65, 5.80).*

***Conclusion**: the study showed unacceptably long total delay. Targeted interventions that enhance TB knowledge and practice, expedite early suspect identification, referral and management of all forms of TB is imperative to reduce total delay in diagnosis and treatment of TB.*

Key words: Tuberculosis, total delay, diagnostic delay, treatment delay, Jimma Zone, Ethiopia

1. Introduction

Tuberculosis (TB) is a major cause of illness in low-resource countries. It is among the top 10 causes of death, and has been the leading cause of death from a single infectious agent in recent years [1,2]. There were an estimated 10.0 million TB cases, and 1.5 million deaths due to TB in 2020[3]. The 30 high TB burden countries are responsible for the majority (86%) of the estimated incident TB cases[2].

Ethiopia is among the high TB burden countries [3]. TB is a major public health problem and one of the leading infectious diseases in Ethiopia. There were an estimated 140 new incident TB cases per 100,000 population in 2020 and TB mortality rate of 19 per 100,000 populations in 2019 in Ethiopia[4]. Ethiopia accounts for 90,000 (3%) of annually missed TB cases worldwide[2,5]. A recent national case detection rate of all forms of TB was 76 percent, which is below the target of World Health Organization[3,6]

Prompt diagnosis and treatment is crucial for efficient TB control program performance. The End TB Strategy indicators can only be realized if diagnosis, treatment, and preventive services for TB are delivered based on the context of universal health coverage, which implies that all people with TB should be early detected and properly treated[2,5]. Delayed identification and diagnosis of TB cases has major role in the transmission of the disease in the community in most high TB burden countries. Early diagnosis and proper treatment of TB will decrease the length of delay in diagnosis and treatment, prevent transmission, increase cure rate, and prevent the development of drug-resistant TB [2,7].

Various studies have been conducted on delays in diagnosis and treatment of TB in different parts of the world [8–15]. A systematic review and meta-analysis conducted in low- and middle-income countries showed that the median total delay ranged from 30 to 366.5 days[16]. Various factors that are associated with diagnostic and treatment delay were identified in earlier studies in Ethiopia and elsewhere. Some of these factors included poor knowledge about TB, first visit to non-formal health providers, long distance to health facility, low level of income, being female, being illiterate, perceived TB stigma, rural residence, having extra-pulmonary TB, patient first visit to lower level health facilities, self-treatment, absence of chest pain, presence of haemoptysis, and >10% body weight loss etc. [8,9,22–27,10,13,15,17–21].

Although various studies were conducted in Ethiopia and elsewhere to assess TB diagnostic and treatment delay, there is limited study to date that has been conducted to define the magnitude and assess associated factors of

total delay in diagnosis and treatment of TB in Jimma Zone, Ethiopia. The length of total delay and associated factors may vary according to the local setting, including the socio-demographic and economic condition of the population in the study area. Understanding the contribution of these factors is important to propose targeted interventions to address diagnostic and treatment delay at the local setting. Therefore, the aim of this study was to determine the length of and identify factors associated with total delay in Jimma Zone, Ethiopia.

2. Materials and methods

2.1. Study setting and design

Analytic cross-sectional study was conducted among all forms of TB patients who started treatment from September 2016 to October 2017. The study was conducted in Jimma Zone, Oromia Region, Southwest Ethiopia (Fig.1). Jimma Zone is located 354 kilometres from Addis Ababa, the capital city of Ethiopia, with a total area of 199,316.18 square kilometres [Jimma Zone health office, 2016]. According to 2017 projected population census, Jimma Zone had an estimated population of 3,261,371, of which 49.9% were women[28]. In 2016, the Zone had 17 districts and two town administrations. A total of seven public hospitals (five were primary, one general and one specialized); 120 health centres, and 494 health posts were registered in the study area during the study period. The hospitals and health centres have been providing TB diagnostic and treatment services. Heath extension workers at health posts have been rendering TB treatment services, screening and referring of TB suspects to the nearest health facilities for confirmatory testing. Non-governmental health facilities such as the Catholic mission and several private clinics were also providing TB diagnostic and treatment services [Jimma Zone health office, 2016; Jimma town health office, 2016].

Fig 1. Map of Jimma Zone, Southwest Ethiopia

2.2. Study population and sampling

All newly diagnosed TB patients were prospectively enrolled from sampled districts' public health facilities and health posts consecutively. Patients whose age was less than 15 years, who could not respond to the interview questions and critically ill patients were excluded from the study. Eight districts and one town administration were

selected from the 17 districts and two town administrations by using a simple random sampling method. Subsequently, all TB DOTS sites in the sampled districts and a town administration were covered by the study. This study is part of a PhD project. It follows a recently published sub-study that compared community- versus facility-based DOTS in a cohort study that enrolled a total of 1,161 study participants[29]. We included all of the 1,161 study participants in the current study. However, in order to address the research objective in the current study, a total sample of 422 calculated using formula for estimating single proportion could have been enough (i.e. considering 50% proportion of delay of more than one month at 95% confidence interval and a margin error of 5%). The inclusion of the 1,161 study participants in the current study is advantageous as it provides more than adequate representative population to address the research questions. The sample size was proportionally allocated to the selected public health facilities based on the previous one year's (before the study's start) patient flow. The study participants were consecutively enrolled until the required sample size was achieved [Fig 2].

Fig 2. Schematic presentation of sampling procedure for a cohort of TB patients, Jimma Zone, 2017

2.3. Data collection and analysis

A structured questionnaire was developed based on the national and WHO's guidelines; and previous studies[30–35]. The questionnaire was translated to the local language (Afan Oromo) by a professional whose mother tongue is the local language. It was checked and peer reviewed for any inconsistencies between the translated version and the original English version of the questions. The translated version of the questionnaire was re-translated to English by another professional who fluently spoke and wrote the local language. Then, it was pre-tested to check for the clarity and applicability of the questionnaire for the context of the study area. Based on the findings of the pre-test, modifications such as clarifying statements were made. We recruited experienced data collectors and supervisors and they were provided with the required training on the data collection and supervision techniques. The whole process of data collection was supervised by the principal investigator. The study participants were consecutively enrolled starting from September 2016 to October 2017. They were all interviewed during the enrolment time.

The collected data were checked for completeness and consistency, coded and entered into the EpiData entry client software, version 4.4.3.1. Then, the data were exported to the statistical package for social sciences software (SPSS) version 21 for analysis. Descriptive statistics were computed for the variables. To assess TB knowledge, a

score of one was given for the correct responses and a score of zero was given for wrong responses. Then the total knowledge score and median score were computed. Those with a total score of less than the median value were categorized as having poor knowledge, while those who scored greater than or equal to the median value were classified as having good TB knowledge. Likewise, the total perceived stigma and median scores were determined. Those with a total score of less than the median value were classified as not having perceived stigma, whereas those who scored greater than or equal to the median value were categorized as having perceived stigma.

The median total delay was used to categorize the sample into delayed and non-delayed patient groups. To analyse the association of independent variables with the dependent variable, we used binary logistic regression analysis. First, bivariate analysis was performed for each independent variable against the respective outcome variable and crude odds ratio (COR) was calculated. Multivariate analysis was conducted comprising candidate variables in the bivariate analysis that scored a p-value of < 0.25 with the outcome variable and the respective adjusted odds ratios (AOR) and 95% confidence intervals (CI) were computed. A p-value < 0.05 was considered statistically significant.

2.4. Operational Definitions

Total delay: the period between onset of TB symptoms and the first start of anti-TB treatment.

Delayed: if the period between the onset of TB symptoms and first start of anti-TB treatment is more than the calculated median total delay (> 35 days).

Medical provider/ formal health care provider: include hospitals, health centres, health posts, private clinics, and drug retail outlets.

Non formal-health providers: traditional healers or herbalists, religious healers

TB Diagnosis

The national guideline for TB, and DR-TB was followed to diagnosis and treat TB [5]. Health care providers identify, triage and examine individuals who present with persistent cough of two or more weeks (any duration for HIV positive), fever for more than two weeks, night sweats, and weight loss of more than 1.5 kg per month. TB diagnosis is made with proper investigations using one or more of the following methods: mycobacteriologcal

examination, chest x-ray, and cytological/histopathology (analysis of body parts/fluids) examinations. Acid-fast-bacilli (AFB) smear microscopy is the most common method used for TB diagnosis and follow up of treatment response. A patient is diagnosed as a pulmonary positive TB case, when he/she has at least one positive result on AFB microscopy, or when his/ her Xpert MTB/RIF test result is positive for mycobacteria. A patient is diagnosed as a pulmonary negative TB case when he/she has signs and symptoms suggestive of TB with at least two negative result on AFB smear microscopy, and secondly, when his/her Xpert MTB/RIF test results detects mycobacterium and a decision to treat with a full course of anti-TB drugs is made. The decision is made based on suggestive findings from supporting laboratory tests and with the aid of proper clinical examination[5].

2.5. Ethical considerations

Ethical clearance was sought from the Regional Committee for Medical Research Ethics (REK Øst), Norway with reference number of 2015/2124 REK sør-øst B. Ethical approval was also obtained from the Institutional Review Board of Jimma University, Ethiopia with reference number of RPGC/389/2016. Written and oral informed consent was secured from each study participant before starting the data collection.

3. Results

3.1. Characteristics of the study participants

A cohort of 1,161 patients participated in this study: 774(66.7%) under FB-DOTS, 387(33.3%) under CB-DOTS) were included. Of these, 51.2% were male, 65.9% were married, and 39.5% were illiterate. The mean (± SD) age for the study participants was 32.2 (±14.41) years, with a range of 15 to 90 years. Nearly half (46.9%) of the patients were 25 - 44 years of age. Of the total study participants, 63.7% were farmers by occupation, 51.7% were rural residents, and 48.8% were smear-positive pulmonary TB patients (Table 1).

Table 1. Socio-demographic and clinical characteristics of the study participants in Jimma Zone, 2017/18 (N=1,161)

Variables		Frequency	Percent (%)
Sex	Male	594	51.2
	Female	567	48.8
Age in year	15 – 24	365	31.4
	25 – 44	544	46.9
	45 – 64	202	17.4
	≥65	50	4.3
	Mean ± SD (32.22±14.41); Minimum 15; Maximum 90; Median 30 (IQR 22, 40)		
Marital status	Single	330	28.4
	Married	765	66.1
	Divorced	30	2.6
	Widowed	34	2.9
Educational level	Illiterate	459	39.5
	Read and write only	98	8.4
	Primary school	396	34.1
	Secondary school	132	11.4
	College/University	76	6.5
Occupation	Farmer	739	63.7
	Merchant	74	6.4
	Government or NGO employee	58	5.0
	Daily laborer	88	7.6
	Housewife	19	1.6
	Student	142	12.2
	Unemployed	41	3.5
Religion	Orthodox Christian	160	13.8
	Muslim	939	80.8
	Protestant	61	5.3
	Catholic	1	0.1
Residence	Town	561	48.3
	Rural	600	51.7
Source of household income	Farming	769	66.2
	Monthly salary	59	5.1
	Private/ trading	78	6.7
	Daily payment	82	7.1
	Family/relative	152	13.1
	Prefer not to answer	21	1.8
Monthly household income in ETB	≤ 1000	584	50.3
	1001- 2500	388	33.4
	2501 - 3500	41	3.5
	> 3500	90	7.8
	Do not have regular income	58	5.0
Previous contact with TB patient	Yes	118	10.2
	No	807	69.5
	Don't know /not sure	236	20.3
Cigarette smoking	Yes	71	6.1
	No	1090	93.9
Drink alcohol	Yes	93	8.0

	No	1068	92.0
Khat chewing	Yes	599	51.6
	No	562	48.4
Previous Rx for TB	Yes	33	2.8
	No	1128	97.2
History of diabetes mellitus	Yes	12	1.0
	No	1149	99.0
Main source of information about TB	Health care providers	914	78.7
	TV/Radio	172	14.8
	Family/relative	53	4.6
	Others	22	1.9
Type of DOTS	Facility-based	774	66.7
	Community-based	387	33.3
TB classification	Smear-positive pulmonary TB	567	48.8
	Smear-negative pulmonary TB	251	21.6
	Extra pulmonary TB	343	29.5
Main mode of TB diagnosis	Bacteriological	592	51.0
	Histopathology/ Biopsy	226	19.5
	Radiological	301	25.9
	Clinical clues	42	3.6
HIV status	Reactive	38	3.3
	Non-reactive	1040	89.6
	Unknown	83	7.1

3.2. Study districts and time to reach the nearest medical provider

A majority (18.9%) of the study participants were from *goma* district. The mean (± SD) time taken to reach the nearest medical provider was 32.86 (±28.52) minutes, ranging from two to 180 minutes (Table 2).

Table 2. Districts and time to reach the nearest medical provider of the study participants at Jimma Zone, 2017/18 (N=1,161)

Variables		Frequency	Percent (%)
District / town administration	*Goma*	220	18.9
	Jimma	157	13.5
	Kersa	122	10.5
	Limu kosa	144	12.4
	Mana	97	8.4
	Omo nada	102	8.8
	Seka chokorsa	120	10.3

	Sokoru	108	9.3
	Tiro afeta	91	7.8
Time to reach nearest medical provider in minutes	≤ 30	792	68.2
	31-60	278	23.9
	> 60	91	7.9
	Mean ± SD (32.86±28.52); Minimum 2; Maximum 180; Median 25 (IQR 15, 45)		

3.3. Patients' first visit to medical providers

A total of 708 (60.98%) patients visited other medical providers (health posts and private clinics) before visiting the current health facility where they got their TB diagnosis and initiated anti-TB treatment. Of these, a majority (169) of the patients who first visited private clinic were not delayed. On the contrary, most (45) of the patients who visited health posts were delayed (Fig 3).

Fig 3. Medical provider first visited for the symptoms with patient delay status at Jimma Zone, 2017/18

3.4. Determinants of total delay

The median total delay was 35(IQR 25, 67 days) days, and for more than half (586) of the patients, the total delay was > 35 days. Variables such as occupation, source of household income, being a smoker, alcohol use, khat chewing, HIV status, TB classification, chest pain, and weight loss were removed from the final model even if these were statistically significant during the bivariate analysis. In the multivariate analysis, patients who had poor knowledge about TB were about four times more likely to be delayed than patients who had good knowledge about TB (AOR=3.92, 95% CI: 2.65, 5.80). Those patients who had swelling or wound in the neck region were three times more likely to be delayed than their counterpart (AOR =3.02, 95% CI: 1.62, 5.62). Patients who attended college or university were 72% less likely to be delayed than those patients who did not read and write (AOR=0.28, 95% CI: 0.10, 0.81). Female patients were about 1.5 times more likely to be delayed than their counterparts (AOR=1.46, 95% CI: 1.00, 2.14). Patients who had a monthly household income of 1001- 2500 ETB had increased total delay compared to patients who earned > 3500 ETB (AOR= 15.75, 95% CI: 2.92, 84.91). Those patients who traveled 30 or less minutes to reach the nearest medical provider were 53% less likely to be delayed than those patients who traveled more than 60 minutes (AOR=0.47, 95% CI: 0.23, 0.94). Patients under CB-DOTS were about 1.5 times

more likely to be delayed than their counterparts (AOR=1.53, 95% CI: 1.02, 2.29). Patients who had no limitation of their day-to-day activity were less likely to be delayed than those patients who had high limitation of their day-to-day activity (AOR= 0.09, 95% CI: 0.01, 0.89) (Table 3).

Table 3. Determinents of total delay among the study partcipants at Jimma Zone,2017/18

**Variables**		**Total delay, No (%)**		**COR (95% CI)**	**AOR (95% CI)**	**P-values**
		Not delayed ≤ 35 days (median)	Delayed > 35 days (median)			
Sex	Male	297 (51.7)	297 (50.7)	1	1	
	Female	278 (48.3)	289 (49.3)	0.96 [0.76,1.21]	1.46 [1.00,2.14]	0.050
Age groups in years	15 - 24	185 (32.2)	180 (30.7)	1	1	
	25 - 44	269 (46.8)	275 (46.9)	0.60 [0.33,1.09]	0.59 [0.20,1.72]	0.331
	45 - 64	102 (17.7)	100 (17.1)	0.63 [0.35,1.14]	0.53 [0.19,1.47]	0.220
	≥ 65	19 (3.3)	31 (5.3)	0.60 [0.32,1.13]	0.49 [0.17,1.39]	0.181
Educational level	Illiterate	177 (30.8)	282 (48.1)	1	1	
	Read and write only	63 (11.0)	35 (6.1)	1.97 [1.21,3.21]	0.74 [0.25,2.19]	0.590
	Primary school	212 (36.9)	184 (31.4)	0.69 [0.37,1.27]	0.41 [0.13,1.27]	0.122
	Secondary school	81 (14.1)	51 (8.7)	1.07 [0.66,1.76]	0.36 [0.13,1.02]	0.053
	College/University	42 (7.3)	34 (5.8)	0.78 [0.44,1.38]	0.28 [0.10,0.81]	0.019
Occupation	Farmer	365 (63.5)	374 (63.8)	1	1	
	Merchant	37 (6.4)	37 (6.3)	0.48 [0.24,0.93]	0.84 [0.18,3.97]	0.820
	Go/NGO employee	33 (5.7)	25 (4.3)	0.46 [0.21,1.03]	0.38 [0.02,7.29]	0.523
	Daily labourer	33 (5.7)	55 (9.4)	0.35 [0.15,0.81]	1.351 [0.07,27.31]	0.845
	House wife	7 (1.2)	12 (2.0)	0.77 [0.35,1.69]	0.56 [0.05,6.78]	0.652
	Student	87 (15.1)	55 (9.4)	0.80 [0.25,2.49]	1.44 [0.19,10.69]	0.722
	Unemployed	13 (2.3)	28 (4.8)	0.29 [0.14,0.62]	0.61 [0.16, 2.27]	0.457
Source of Household income	Farming	380 (66.1)	389 (66.4)	1	1	
	Monthly salary	36 (6.3)	23 (3.9)	6.14 [1.80,21.02]	0.17 [0.02,1.54]	0.114
	Private/trading	40 (7.1)	38 (6.5)	3.83 [1.01,14.49]	0.14 [0.01,1.54]	0.107
	Daily payment	28 (4.9)	54 (9.2)	5.70 [1.55,20.92]	0.37 [0.04,3.58]	0.387
	Family/relative	73 (12.7)	79 (13.5)	11.57 [3.14,42.66]	0.50 [0.05,4.95]	0.552
	Prefer not to answer	18 (3.1)	3 (0.5)	6.49 [1.84,22.96]	0.39 [0.05,3.31]	
Monthly Household income in ETB	≤ 1000	301 (52.3)	283 (48.3)	5.88 [2.74,12.61]	9.51 [1.82,49.72]	0.008
	1001- 2500	162 (28.2)	226 (38.6)	8.72 [4.02,18.89]	15.75 [2.92,84.91]	0.001
	2501- 3500	23 (4.0)	18 (3.1)	4.89 [1.86,12.88]	6.92 [1.03,46.58]	0.047
	> 3500	39 (6.8)	51 (8.7)	1	1	
	No regular income	50 (8.7)	8 (1.4)	8.17 [3.48,19.22]	13.29 [2.27,77.65]	0.004
Time to reach nearest medical provider in minute	≤ 30	391 (68.0)	401 (68.4)	0.77[0.49,1.19]	0.47[0.23,0.94]	0.034
	31- 60	145(25.2)	133(22.7)	0.69[0.43,1.12]	0.68[0.31,1.46]	0.317
	> 60	39(6.8)	52(8.9)	1	1	
Cigarette smoking	Yes	25 (4.3)	46 (7.8)	1.87 [1.14,3.09]	1.22 [0.54,2.75]	0.627
	No	550 (95.7)	540 (92.2)	1	1	
Alcohol use	Yes	36 (6.3)	57 (9.7)	1.61[1.05,2.49]	1.01 [0.49,2.08]	0.985
	No	539 (93.7)	529 (90.3)	1	1	
Khat chewing	Yes	320 (55.7)	279 (47.6)	0.72 [0.58,0.91]	0.83 [0.56,1.24]	0.356
	No	255 (44.3)	307(52.4)	1	1	
HIV status	Reactive	20 (3.5)	18 (3.1)	1	1	

	Non-reactive	525 (91.3)	515 (87.9)	0.51[0.23,1.11]	0.98 [0.28,3.45]	0.970
	Unknown	30 (5.2)	53 (9.0)	0.56 [0.35,0.88]	0.96 [0.47,1.98]	0.917
Current illness status	No limitation of day-to-day activity	362 (63.0)	229 (39.1)	0.25 [0.05,1.31]	0.09 [0.01,0.89]	0.040
	Slight limitation	211(36.7)	352(60.0)	0.67 [0.13,3.47]	0.22 [0.02,2.12]	0.189
	High limitation	2(0.3)	5(0.9)	1	1	
Type of DOTS	Facility-based	360 (62.6)	414 (70.6)	1	1	
	Community-based	215 (37.4)	172 (29.4)	1.44 [1.13,1.84]	1.53 [1.02,2.29]	0.041
TB classification	Smear-positive PTB	306 (53.2)	221 (44.5)	1	1	
	Smear-negative PTB	131(22.8)	120 (20.5)	0.57 [0.44,0.75]	0.78 [0.48,1.26]	0.309
	Extra PTB	138(24.0)	205(35.0)	0.62 [0.44,0.86]	1.07 [0.64,1.79]	0.798
Medical provider first visited for symptoms	Health post	7 (2.0)	45 (12.4)	1	1	
	Health centre	111(32.1)	128 (35.4)	3.21[0.49,20.96]	2.23 [0.25,19.50]	0.470
	Hospital	57 (16.5)	75 (20.7)	0.58 [0.10,3.21]	0.79 [0.12,5.86]	0.814
	Private clinic	169 (48.8)	110(30.4)	0.66 [0.12,3.72]	0.49 [0.07,3.77]	0.499
	Other	2 (0.6)	4 (1.1)	0.33 [0.06,1.81]	0.39 [0.05,2.89]	0.356
Chest pain	Yes	395 (68.7)	328 (56.0)	1	1	
	No	180 (31.3)	258 (44.0)	0.58 [0.46,0.74]	0.75 [0.50,1.13]	0.172
Haemoptysis	Yes	83 (14.4)	75 (12.8)	0.87 [0.62,1.22]	1.24 [0.68,2.27]	0.479
	No	492 (85.6)	511 (87.2)	1	1	
Weight loss (10%)	Yes	372 (64.7)	345 (58.9)	0.78 [0.62,0.99]	1.28 [0.86,1.91]	0.217
	No	203 (35.3)	241(41.1)	1	1	
Swelling or wound around neck region	Yes	42 (7.3)	106(18.1)	2.80[1.92,4.09]	3.02 [1.62,5.62]	< 0.001
	No	533 (92.7)	480(81.9)	1	1	
Knowledge about TB	Poor	130 (22.6)	333 (56.8)	4.51[3.49,5.81]	3.92 [2.65,5.80]	< 0.001
	Good	445 (77.4)	253 (43.2)	1	1	
Perceived to be stigmatized	No	183 (31.8)	183 (31.2)	0.97 [0.76,1.25]	1.40 [0.89,2.20]	0.143
	Yes	392 (68.2)	403 (68.8)	1	1	

AOR = adjusted odds ratio; COR = crude odds ratio; CI = confidence interval. 1= Reference group

4. Discussion

In this study, we assessed the length and associated factors of total delay among TB patients. A systematic review and meta-analysis conducted in low- and middle-income countries reported that the median total delay ranged from 30 to 366.5 days [16].We observed a median total delay of 35 days which is similar to the study done in Addis Ababa, Ethiopia[36] , but lower than the findings reported from earlier studies conducted in different Regions of Ethiopia [17,19,22,24,37]. The shorter total delay observed in the present study may be related to several reasons: these may include improved access to TB diagnostic and treatment facilities through decentralization of health services to the community, and health extension workers' contribution in identifying suspected TB cases and referring them to health centers for smear microscopy test[38].

Even though the observed total delay in our study was relatively lower compared to earlier study findings in Ethiopia, it is still long delay given the need for immediate diagnosis and treatment initiation for TB patients. In a recent qualitative study conducted in the study area, different barriers were identified that may have implications for increased total delay. Among these were shortage of resources including basic infrastructure and limited TB diagnostic services; and delays in the diagnostic process at the health facilities[39]. Solving these barriers through targeted interventions is crucial to reduce the observed relatively long total delay. Long delays in diagnosis and treatment initiation increase the severity and complications of disease that may result in unfavorable treatment outcome[22,40]. It also increases the risk of developing drug resistant TB which leads to treatment failure, high mortality rates, and high transmission rate of drug resistant TB[41,42]. These findings emphasize the importance of early diagnosis and prompt treatment of TB. Conversely, the observed median total delay in the current study is higher than the study findings in other parts of Ethiopia that reported 23 days and 33 days[43,44]. This discrepancy might be because of differences in the study settings i.e. socio-economic status, study population, access to TB diagnostic facilities, and utilization of health care services among the population.

The analysis of socio-demographic, economic, and clinical factors revealed significant associations with increased total delay. In line with our study, low level of education [16,17,19,37], long distance [13,19], and poor knowledge about TB[19,22,24] were identified as predictors of total delay in former studies in Ethiopia and elsewhere. There is a synergetic effect between educational status and knowledge about TB. Our findings underscored that educational level is an important determinant of total delay. A former study in Ethiopia revealed that individuals with a higher level of education had better knowledge about TB compared to those who did not read and write[45]. Our study showed that the educational level of the patients may not directly be a causative factor for the delay but do significantly affect the knowledge level of the study participants. A study from the Gambia showed an association of educational level with knowledge, attitude and health-seeking behavior regarding TB[46]. Another study from Malaysia reported that TB education program at school was an effective intervention for improvement in the mean score of knowledge, preventive practice, and perceived stigma about TB[47]. In addition, a study conducted in Ethiopia indicated that there was association between educational level and knowledge about TB in which having good knowledge of TB led to a more positive attitude and better practice in relation to TB prevention and control[48]. This was supported by findings from a qualitative meta-synthesis conducted in Nigeria which reported that low education level and poor knowledge about TB were major barriers to TB diagnosis and

treatment[14]. Compared to the uneducated persons, individuals with better educational level are more likely to have access to better economic situation which consecutively improves their access to health information and care [43,45]. Moreover, the level of their knowledge about TB influence their perceptions about TB and change their health care-seeking behaviour[43,45].

We observed a strong association between having swelling or wound in the neck region and long total delay. Cervical TB lymphadenitis is one of the most common manifestations of extra-pulmonary TB which contributes to diagnostic delay. The common sign and symptom is a chronic, painless swelling in the neck region (no other remarkable symptom). Thus, the diagnosis of this type of TB requires a high index of suspicion and use of different diagnostic methods. Practically, it is not possible to apply all diagnostic procedures for all symptomatic patients. In this regard, ruling out for other causes of neck mass could contribute to diagnostic delay. Because of the absence of culture and pathology services in most health centers in Ethiopia including the study area, it is difficult to early diagnose such type of TB[49,50]. A study from Zanzibar showed that from all patients with long total delay (> 6 months), a majority (90%) were patients with TB lymphadenitis[51]. Extra-pulmonary TB cases are seldom infectious as they are considered to have less contribution in TB transmission. However, delay in starting treatment for the patients may result in disseminated TB and increased mortality [50,51]. As peripheral health care facilities in the study area do not have the capacity to diagnose extra-pulmonary TB cases, it is important to develop diagnostic algorithms for timely referral of presumptive extra-pulmonary TB patients. Therefore, training is essential for the health extension workers so that they can screen TB lymphadenitis suspects in the community for prompt referral to the next level of health care for better diagnosis and treatment [52].

Our study revealed that women were more likely to have long total delay compared to men. This finding is consistent with former studies conducted in Ethiopia and elsewhere [19,53,54]. Economic status, cultural beliefs, and perceived stigma are barriers of early care seeking for most female patients[55]. Women in high burden countries like Ethiopia experienced long delays in TB diagnosis and treatment because of challenges related to TB services [56]. Most women faced problems in accessing TB service because of resource limitations, power imbalances, and poor knowledge about TB[56]. Women who have less access to quality healthcare, and delayed to seek formal health care were prone to long delays [55]. This may partly be related to the fact that women have the main responsibility for domestic tasks and care for their family members, especially children and elders. Women often lack economic independence, have less time and are less empowered compared to men in regards to care for

themselves[19]. Therefore, it is important to recognize sex differences in individual's TB care-seeking. Targeted interventions that enhance women empowerment are required to improve poor health outcomes among women. Socio-economic support interventions could change their health seeking behaviour, improve adherence and treatment outcome for TB patients who are women [57,58]. In addition, gender-based health education and behavioural change communication (BCC) interventions focusing on early health seeking counselling are required to reduce the burden of TB among women. BCC strategies are efficient, effective, sustainable and acceptable because messages can tailored to gender- or age-based target groups [59,60].

Monthly household income was another determinant variable that was significantly associated with total delay. This finding is in line with previous study results from Ethiopia[43,61,62] and Pakistan[12]. This could be due to the fact that individuals who did not have adequate monthly income could not be able to seek health care early because of several reasons such as unaffordable medical costs and transport expenses[43,62]. Therefore, they are likely to be prone to delayed diagnosis and treatment start. In resource limited settings like Ethiopia where there are several traditional practices and low access to quality health service, patients commonly seek health care from informal medical providers, as a result the patients might get traditional treatment that could delay them from timely seeking medical care from formal medical providers. TB diagnosis and treatment services in Ethiopia is given for free, money is mostly needed for covering costs for transportation, accommodation and food for the patient. Individuals who have low level of income may need to work long hours, as a result they may not have time to early seek health care when they get ill. Patients with inadequate income are more likely to visit formal-health care provider only when they become critically ill[43,62]. A systematic review conducted in high TB burden settings reported that livelihood, work, and family were given priority which led them to have a long delay in health care-seeking[56]. This finding shows the importance of improving the socio-economic condition of the population for effective TB control. As per the Sustainable Development Goals (SDG), the world is committed to end poverty by 2030[63,64]. According to a recent World Bank report, the poverty rate in Ethiopia fell from 44 percent in 2000 to 21 percent in 2018[65,66]. If this trend continues, the contribution of poverty to TB disease and transmission may be significantly reduced in Ethiopia including the study area.

Time taken to reach the nearest medical provider was associated with total delay. Long distance traveling to reach the nearest health facility was also reported in previous studies conducted in Ethiopia and other countries as barrier to individuals' health-seeking behavior which leads to lengthening of patient's delay [11,67–71]. A recent

qualitative study from the study area identified that long distance traveling to get TB diagnostic services was a barrier for most TB patients from the rural settings[39]. This could also be related with patients who were under FB-DOTS travel longer distance and more delayed than those patients under FB-DOTS.

Our study showed that patients under CB-DOTS were more likely to be delayed than those patients under FB-DOTS. This could be due to the fact that patients under CB-DOTS were rural residents and most of patients under FB-DOTS were urban residents. Rural residence was reported as independent predictor of delay in a systematic review conducted in Ethiopia[72]. One of the reasons might be that rural residents had poor access to health information and TB diagnostic and treatment facilities compared to urban residents. This might be due to the scarcity of formal health care providers in rural area of Ethiopia. Thus, patients from rural areas might take long chains of care-seeking through informal health care providers[56,73]. A previous study from Southwest Ethiopia revealed that proportion of households sought care from formal health care providers was lower among rural compared to urban households[74]. Moreover, rural residents lack regular health information about the disease and do not seek health care early compared to the urban residents [72,75]. In addition, rural residency makes it hard to travel to diagnostic and treatment facilities due to absence of road or distance (as long distance travelling discourages initiation of tuberculosis treatment)[72,75].

This study has strengths and limitations. The strength of the study is that we included a relatively larger sample size compared to sample sizes used in other similar studies in Ethiopia. Thus, it provides more than adequate representative population to address the research questions. With regards to potential limitations, the study was only carried out in government health facilities; hence the findings cannot be generalized to all TB patients in the study area. There are also several private health care facilities and non-formal health care providers whereby TB patients may seek health care for their symptoms. Moreover, the present study focused on adult TB patients aged > 15 years who were treated at public health facilities; therefore, the results among other age groups and in other similar settings during the study period might be different. In addition, the reported duration of symptoms and first visit for treatment seeking is based on patients' ability to recall and interpretation of their symptoms. Since patients might not exactly remember the exact date of start of their symptoms and first visit to a medical provider, it is subject to a recall bias. However, we have tried to reduce the recall bias by using different techniques such as using local calendars linking to major religious and national days to explain their perceived date of onset of TB symptoms and first visit to a medical provider.

5. Conclusion

The study showed unacceptably long total delay in diagnosis and treatment start for TB patients in the study area. The study also revealed important factors associated with total delay. Poor knowledge of TB, swelling or wound around the neck region, being women and low level of education were identified as factors associated with total delay. Several interventions are required to reduce the observed length of total delay. Efficient implementation strategies for early case detection and treatment initiation should be practiced to shorten total delay. Among others, well-crafted IEC and BCC strategies on TB need to be designed to increase awareness and health service utilization among the population in the study area. Swelling or wound around the neck region in a TB patient may be linked to TB lymphadenitis or disseminated TB. The health care facilities at peripheral level in the study area do not have the capacity to diagnose TB lymphadenitis cases. Thus, it is important to develop diagnostic algorithms for timely detection and referral of presumptive TB lymphadenitis patients to the nearest medical providers with the capacity to diagnose TB lymphadenitis. Then, the delay period among this group of patients is shortened and prompt treatment is initiated on time.

Acknowledgments

Our gratitude goes to the University of Oslo, Institute of Health and Society and Jimma University for supporting us to undertake this study. We would like to thank the Oromia Health Bureau, the Jimma Zone and Jimma Town health offices and all the study participants for providing us with the necessary information.

Authors’ contributions

BME initiated the study, wrote the proposal, and conducted the data collection, data analysis, and writing this manuscript. MS contributed to the conception and proposal of the study, and contributed to the writing of this manuscript by critically reviewing it. CG contributed to the writing of this manuscript by critically reviewing it. SAY contributed to the conception and proposal of the study, analysis and interpretation of the data. He also contributed to the manuscript writing by critically reviewing it. All the authors read and approved the final manuscript.

Data reporting

All data analyzed during the current study are available from the corresponding author on request.

Authors' information

BME (MSc, Assistant Professor, PhD candidate) is working at Jimma University, Ethiopia as instructor; MS (PhD, Professor) is working at department of nursing and health promotion, faculty of health sciences, Oslo Met – Oslo Metropolitan University, Norway as a researcher; CG (PhD, Professor) is working at department of community medicine and Global health, University of Oslo, Norway as instructor and head of the department; SAY (PhD, Associate professor) is working at Coalition for Epidemic Preparedness Innovations (CEPI), Oslo, Norway; and at Institute of Clinical Medicine, University of Oslo, Norway.

References

1. World Health Organization. Global Tuberculosis Report. France; 2019.
2. World Health Organization. Global tuberculosis report 2020. Geneva; 2020.
3. World Health Organization. Global Tuberculosis Report 2021. Geneva: World; 2021.
4. Ministry of Health Ethiopia. ANNUAL PERFORMANCE REPORT 2020/2021. Addis Ababa, Ethiopia; 2020.
5. Federal Democratic republic of Ethiopia Ministry of Health. National Guidelines for TB, DR-TB and Leprosy in Ethiopia. Sixth Edit. Addis Ababa, Ethiopia; 2018.
6. Ministry of Health Ethiopia. HEALTH & HEALTH RELATED INDICATORS 2020/2021. Addis Ababa, Ethiopia; 2020.
7. World Health Organization (WHO). Global Tuberculosis Report. France; 2018.
8. Peri AM, Bernasconi DP, Galizzi N, Matteelli A, Codecasa L, Giorgio V, et al. Determinants of patient and health care services delays for tuberculosis diagnosis in Italy : a cross-sectional observational study. BMC infectious diseases. 2018;18.
9. Nogueira B, Rolla C, Akrami KM, Kiene SM. Factors associated with tuberculosis treatment delay in patients co-infected with HIV in a high prevalence area in Brazil. PLoS ONE. 2018;13: 1–14. Available: https://doi.org/10.1371/ journal.pone.0195409%0A
10. Owolabi OA, Jallow AO, Jallow M, Sowe G. Delay in the diagnosis of pulmonary tuberculosis in The Gambia, West Africa: A cross-sectional study. International Journal of Infectious Diseases. 2020;101: 102–106. Available: http://creativecommons.org/licenses/by-nc- nd/4.0
11. Said K, Hella J, Mhalu G, Chiryankubi M, Masika E, Maroa T, et al. Diagnostic delay and associated factors

among patients with pulmonary tuberculosis in Dar es Salaam, Tanzania. Infectious Diseases of Poverty. 2017;6. doi:10.1186/s40249-017-0276-4

12. Saqib SE, Ahmad MM, Amezcua-prieto C. Treatment Delay among Pulmonary Tuberculosis Patients within the Pakistan National Tuberculosis Control Program. AmJTropMedHyg. 2018;99: 143–149. doi:10.4269/ajtmh.18-0001

13. Nguyen HT, Duong B, Beach L. Delays in the diagnosis and treatment of tuberculosis patients in Vietnam: A cross-sectional study. BMC Public Health. 2007;7. doi:10.1186/1471-2458-7-110

14. Oga-omenka C, Wakdet L, Menzies D, Zarowsky C. A qualitative meta-synthesis of facilitators and barriers to tuberculosis diagnosis and treatment in Nigeria. BMC Public Health. 2021;21. doi:https://doi.org/10.1186/s12889-021-10173-5

15. Takarinda KC, Harries AD, Nyathi B, Ngwenya M, Mutasa-apollo T, Sandy C. Tuberculosis treatment delays and associated factors within the Zimbabwe national tuberculosis programme. BMC Public Health. 2015;15: 1–12. doi:10.1186/s12889-015-1437-7

16. Getnet F, Demissie M, Assefa N, Mengistie B, Worku A. Delay in diagnosis of pulmonary tuberculosis in low-and middle-income settings: Systematic review and meta-analysis. BMC Pulmonary Medicine. 2017;17. doi:10.1186/s12890-017-0551-y

17. Asefa A, Teshome W. Total Delay in Treatment among Smear Positive Pulmonary Tuberculosis Patients in Five Primary Health Centers , Southern Ethiopia : A Cross Sectional Study. PLoS ONE. 2014;9. doi:10.1371/journal.pone.0102884

18. Asres A, Jerene D, Deressa W. Delays to treatment initiation is associated with tuberculosis treatment outcomes among patients on directly observed treatment short course in Southwest Ethiopia : a follow-up study. BMC Pulmonary Medicine. 2018;18. doi:https://doi.org/10.1186/s12890-018-0628-2

19. Awoke N, Dulo B, Wudneh F. Total Delay in Treatment of Tuberculosis and Associated Factors among New Pulmonary TB Patients in Selected Health Facilities of Gedeo Zone , Southern Ethiopia , 2017 / 18. interdisciplanry persepctive on infectious disease. 2019;2019. doi:10.1155/2019/2154240

20. Bogale S, Diro E, Shiferaw AM, Yenit MK. Factors associated with the length of delay with tuberculosis diagnosis and treatment among adult tuberculosis patients attending at public health facilities in Gondar town, Northwest, Ethiopia. BMC Infectious Diseases. 2017;17. doi:10.1186/s12879-017-2240-0

21. Evenden P, Roche A, Karo B, Balasegaram S, Anderson CS. Presentation and healthcare delays among people with tuberculosis in London , and the impact on treatment outcome. BMJ Open Respiratory Resaerch. 2019;6: 1–11. doi:10.1136/bmjresp-2019-000468

22. Gebreegziabher SB, Bjune GA, Yimer SA. Total Delay Is Associated with Unfavorable Treatment Outcome among Pulmonary Tuberculosis Patients in West Gojjam Zone , Northwest Ethiopia : A Prospective Cohort Study. PLoS ONE. 2016;11. doi:10.1371/journal.pone.0159579

23. Gebreegziabher SB, Bjune GA, Yimer SA. Patients ' and health system ' s delays in the diagnosis and treatment of new pulmonary tuberculosis patients in West Gojjam Zone , Northwest Ethiopia : a cross-sectional study. BMC Infectious Diseases. 2016;16. doi:10.1186/s12879-016-1995-z

24. Hamza A, Demissie M, Gare S, Teshome G. Delay in Tuberculosis Diagnosis among Tuberculosis Patients at the Three Hospitals : Asella , Robe and Abomsa of Arsi Zone , Oromia Regional State , March , 2015. Open Access Library Journal. 2015;2: 1–13. doi:10.4236/oalib.1101947

25. Seid A, Metaferia Y. Factors associated with treatment delay among newly diagnosed tuberculosis patients in Dessie City and surroundings, Northern Central Ethiopia: A cross-sectional study. BMC Public Health. 2018;18: 1–13. doi:10.1186/s12889-018-5823-9

26. Shiferaw MB, Zegeye AM. Delay in tuberculosis diagnosis and treatment in Amhara state , Ethiopia. BMC health services research. 2019;19.

27. Wyk SS Van, Enarson DA, Beyers N, Lombard C, Hesseling AC. Consulting private health care providers aggravates treatment delay in urban South African tuberculosis patients. Int J Tuber Lung Dis,. 2011;15: 1069–1076. doi:10.5588/ijtld.10.0615

28. Central Statistical Authority. 2007 POPULATION and HOUSING CENSUS OF Ethiopia. Addis Ababa, Ethiopia; 2012.

29. Ereso BM, Sagbakken M, Gradmann C, Yimer SA. Treatment outcomes of patients with drug-sensitive tuberculosis under community- based versus facility-based directly observed treatment , short course strategy in Southwest Ethiopia : a prospective cohort study. BMJ Open. 2021;11. doi:10.1136/bmjopen-2020-048369

30. Boogaard J Van Den, Lyimo R, Irongo CF, Boeree MJ, Schaalma H, Aarnoutse RE, et al. Community vs . facility-based directly observed treatment for tuberculosis in Tanzania ' s Kilimanjaro Region. Int J Tuber Lung Dis,. 2009;13: 1524–1529.

31. Datiko DG, Lindtjørn B. Health Extension Workers Improve Tuberculosis Case Detection and Treatment Success in Southern Ethiopia : A Community Randomized Trial. 2009;4: e5443. doi:10.1371/journal.pone.0005443

32. Federal MoH Ethiopia. Guidelines for clinical and programmatic management of TB, leprosy and TB/HIV in Ethiopia. 6th Editio. Addis Ababa, Ethiopia; 2016.

33. World Health Organization (WHO). Treatment of tuberculosis, Guidlines for treatment of drug susceptible tuberculosis and patient care [Internet]. Geneva, Switzerland; 2017. Available: http://apps.who.int/iris.

34. Yassin MA, Datiko DG, Tulloch O, Markos P, Aschalew M, Shargie EB, et al. Innovative Community-Based Approaches Doubled Tuberculosis Case Notification and Improve Treatment Outcome in Southern Ethiopia. PLoS ONE. 2013;8: e63174. doi:10.1371/journal.pone.0063174

35. Datiko DG, Lindtjørn B. Cost and Cost-Effectiveness of Treating Smear-Positive Tuberculosis by Health Extension Workers in Ethiopia : An Ancillary Cost-Effectiveness Analysis of Community Randomized Trial. PLoS ONE. 2010;5: e9158. doi:10.1371/journal.pone.0009158

36. Adenager GS, Alemseged F, Asefa H, Gebremedhin AT. Factors Associated with Treatment Delay among Pulmonary Tuberculosis Patients in Public and Private Health Facilities in Addis Ababa , Ethiopia. Tuberclosis Research and Treatment. 2017;2017. doi:10.1155/2017/5120841

37. Hussen A, Biadgilign S, Tessema F, Mohammed S, Deribe K, Deribew A. Treatment delay among pulmonary tuberculosis patients in pastoralist communities in Bale Zone , Southeast Ethiopia. BMC Resaerch Note. 2012;5. Available: http://www.biomedcentral.com/1756-0500/5/320

38. Datiko DG, Yassin MA, Theobald SJ, Blok L, Suvanand S, Creswell J, et al. Health extension workers improve tuberculosis case finding and treatment outcome in Ethiopia : A large-scale implementation study. BMJ Global Health. 2017;2: 1–8. doi:10.1136/bmjgh-2017-000390

39. Ereso BM, Yimer SA, Gradmann C, Sagbakken M. Barriers for tuberculosis case finding in Southwest Ethiopia : A qualitative study. PLoS ONE. 2020;15: 1–20. doi:10.1371/journal.pone.0226307

40. Virenfeldt J, Rudolf F, Camara C, Furtado A, Gomes V, Aaby P, et al. Treatment delay affects clinical severity of tuberculosis : a longitudinal cohort study. BMJ Open. 2014;4. doi:10.1136/bmjopen-2014-004818

41. Kuznetsov VN, Grjibovski AM, Mariandyshev AO, Johansson E, Bjune GA. Two vicious circles contributing to a dignostic delay for tuberculosis patients in Arkhangelsk. Emerging Health Threats J.

2014;7. doi:10.3402/ehtj.v7.24909

42. Mahato RK, Laohasiriwong W, Vaeteewootacharn K. Major Delays in the Diagnosis and Management of Tuberculosis Patients in Nepal. Journal of clinical and dignostic resaerch. 2015;9: 5–9. doi:10.7860/JCDR/2015/16307.6633

43. Ayalew YE, Fikadu Ambaw Yehualashet WAB, Gobeza MB. Delay for Tuberculosis Treatment and Its Predictors among Adult Tuberculosis Patients at Debremarkos Town Public Health. Tuberclosis Research and Treatment. 2020;2020. doi:doi.org/10.1155/2020/1901890

44. Datiko DG, Jerene D, Suarez P. Patient and health system delay among TB patients in Ethiopia : Nationwide mixed method cross-sectional study. BMC Public Health. 2020;20. doi:doi.org/10.1186/s12889-020-08967-0

45. Daniel G Datiko, Habte D, Jerene D, Suarez P. Knowledge , attitudes , and practices related to TB among the general population of Ethiopia : Findings from a national cross-sectional survey. PLoS ONE. 2019;14: e0224196. doi:doi.org/ 10.1371/journal.pone.0224196

46. Bashorun AO, Linda C, Omoleke S, Kendall L, Donkor SD, Kinteh M, et al. Knowledge , attitude and practice towards tuberculosis in Gambia : a nation-wide cross-sectional survey. BMC Public Health. 2020;20. doi:https://doi.org/10.1186/s12889-020-09685-3

47. Idris NA, Rosnani Z, Rosediani M, Rosmawati NH, Azlina I, Zahiruddin WM. The Effectiveness of Tuberculosis Education Programme in Kelantan , Malaysia on Knowledge , Attitude , Practice and Stigma Towards Tuberculosis among Adolescents. Malays J Med Sci. 2020;27: 102–114. doi:https://doi.org/10.21315/mjms2020.27.6.10

48. Adane K, Spigt M, Johanna L, Noortje D, Abera SF, Dinant GJ. Tuberculosis knowledge, attitudes, and practices among northern Ethiopian prisoners: Implications for TB control efforts. PLoS ONE. 2017;12. doi:10.1371/journal.pone.0174692

49. Deveci HS, Kule M, Kule ZA, Habesoglu TE. Diagnostic challenges in cervical tuberculous lymphadenitis: A review. North Clin Istanbul. 2016;3: 150–155. doi:10.14744/nci.2016.20982

50. Tagne Nouemssi AB. Disseminated tuberculous lymphadenitis presenting as cervical mass in patient with HIV infection , worsening after antiretroviral initiation : diagnosis and treatment challenges. BMJ Case Rep. NY, United states of America; 2018. doi:10.1136/bcr-2017-221775

51. Jørstad MD, Aßmus J rg, Marijan M, Sviland L, Mustafa T. Diagnostic delay in extrapulmonary tuberculosis and impact on patient morbidity : A study from Zanzibar. PLoS ONE. 2018;13: e0203593. doi:https://doi.org/10.1371/journal. pone.0203593

52. Biadglegne F, Tesfaye W, Sack U, Rodloff AC. Tuberculous Lymphadenitis in Northern Ethiopia : In a Public Health and Microbiological Perspectives. PLoS ONE. 2013;8: e81918. doi:10.1371/journal.pone.0081918

53. Krishnan L, Akande T, Shankar A V, Mcintire KN, Gounder CR, Gupta A, et al. Gender-Related Barriers and Delays in Accessing Tuberculosis Diagnostic and Treatment Services : A Systematic Review of Qualitative Studies. Tuberclosis Research and Treatment. 2014;2014. doi:10.1155/2014/215059

54. Lee C, Wang J, Lin H, Lin P, Chang J, Suk C, et al. Treatment delay and fatal outcomes of pulmonary tuberculosis in advanced age : a retrospective nationwide cohort study. BMC Infectious Diseases. 2017;17. doi:10.1186/s12879-017-2554-y

55. Hargreaves JR, Boccia D, Evans CA, Adato M, Petticrew M. The Social Determinants of Tuberculosis : From Evidence to Action. American Journal of Public Health. 2011;101: 654–662. doi:10.2105/AJPH.2010.199505

56. Teo KAJ, Singh SR, Prem K, Hsu LY, Yi S. Duration and determinants of delayed tuberculosis diagnosis and treatment in high ‐ burden countries : a mixed ‐ methods systematic review and meta ‐ analysis. Respiratory Research. 2021;22. doi:10.1186/s12931-021-01841-6

57. Hoorn R Van, Jaramillo E, Collins D, Gebhard A, Hof S Van Den. The Effects of Psycho-Emotional and Socio- Economic Support for Tuberculosis Patients on Treatment Adherence and Treatment Outcomes – A Systematic Review and Meta- Analysis. PLoS ONE. 2016;11: e0154095. doi:10.1371/journal.pone.0154095

58. Peddireddy V. Quality of Life , Psychological Interventions and Treatment Outcome in Tuberculosis Patients : The Indian Scenario Psychological Distress in Tuberculosis. Frontiers in Psychology. 2016;7. doi:10.3389/fpsyg.2016.01664

59. López-alcalde J, Stallings E, Nunes SC, Chávez AF, Daheron M, Cosp XB, et al. Consideration of sex and gender in Cochrane reviews of interventions for preventing healthcare-associated infections : a methodology study. BMC Health Services Research. 2019;19. doi:https://doi.org/10.1186/s12913-019-4001-9

60. Ngigi S, Busolo DN. Behaviour Change Communication in Health Promotion : Appropriate Behaviour Change Communication in Health Promotion : Appropriate Practices and Promising Approaches. International Journal of Innovative Research and Development ·. 2018;7. doi:10.24940/ijird/2018/v7/i9/SEP18027

61. Wondawek TM, Ali MM. Delay in treatment seeking and associated factors among suspected pulmonary tuberculosis patients in public health facilities of Adama town , eastern Ethiopia. BMC Public Health. 2019;19. doi:doi.org/10.1186/s12889-019-7886-7

62. Bogale S, Diro E, Shiferaw AM, Yenit MK. Factors associated with the length of delay with tuberculosis diagnosis and treatment among adult tuberculosis patients attending at public health facilities in. BMC infectious diseases. 2017;17: 1–10. doi:10.1186/s12879-017-2240-0

63. Tulder R Van, Rodrigues SB. The UN ' s Sustainable Development Goals : Can multinational enterprises lead the Decade of Action ? Journal of International Business Policy. 2021;2021. doi:10.1057/s42214-020-00095-1

64. GBD 2017 SDG Collaborators. Measuring progress from 1990 to 2017 and projecting attainment to 2030 of the health-related Sustainable Development Goals for 195 countries and territories : a systematic analysis for the Global Burden of Disease Study 2017. Lancet. 2018;392: 2091–2138. doi:10.1016/S0140-6736(18)32281-5

65. World Bank. Poverty Overview: Development news resaerch data [Internet]. 2021 [cited 26 Dec 2021]. Available: https://www.worldbank.org/en/topic/poverty/overview#1

66. Thelwell K. SUCCESSFUL DEVELOPMENT: REDUCING POVERTY IN ETHIOPIA [Internet]. 2019 [cited 26 Dec 2021]. Available: https://borgenproject.org/tag/poverty-in-ethiopia/

67. Huong NT, Vree M, Duong BD, Khanh VT, Loan VT, Co N V., et al. Delays in the diagnosis and treatment of tuberculosis patients in Vietnam: A cross-sectional study. BMC Public Health. 2007;7: 1–8. doi:10.1186/1471-2458-7-110

68. Fluegge K, Malone LL, Nsereko M, Okware B, Wejse C, Kisingo H, et al. Impact of geographic distance on appraisal delay for active TB treatment seeking in Uganda: A network analysis of the Kawempe Community Health Cohort Study. BMC Public Health. 2018;18. doi:10.1186/s12889-018-5648-6

69. Ukwaja KN, Alobu I, Nweke CO, Onyenwe EC. Healthcare-seeking behavior, treatment delays and its determinants among pulmonary tuberculosis patients in rural Nigeria: A cross-sectional study. BMC health services research. 2013;13: 1–9. doi:10.1186/1472-6963-13-25

70. Mesfin MM, Newell JN, Walley JD, Gessessew A, Madeley RJ. Delayed consultation among pulmonary tuberculosis patients: A cross sectional study of 10 DOTS districts of Ethiopia. BMC Public Health. 2009;9:

1–10. doi:10.1186/1471-2458-9-53

71. Getnet F, Hashi A, Mohamud S, Mowlid H, Klinkenberg E. Low contribution of health extension workers in identification of persons with presumptive pulmonary tuberculosis in Ethiopian Somali Region pastoralists. BMC Health Services Research. 2017;17: 1–9. doi:10.1186/s12913-017-2133-3

72. Obsa SM, Binu WD, Wosene NG, Gebremedhin D, Chemeda D, Edosa, et al. Treatment seeking delay and associated factors among tuberculosis patients attending health facility in Ethiopia from 2000 to 2020 : A systematic review and meta analysis. PLoS ONE. 2021;16: e0253746. doi:10.1371/journal.pone.0253746

73. Alene M, Assemie MA, Yismaw L, Gedif G, Ketema DB. Patient delay in the diagnosis of tuberculosis in Ethiopia : a systematic review and meta-analysis. BMC Infectious Diseases. 2020;20. doi:doi.org/10.1186/s12879-020-05524-3

74. Begashaw B, Tessema F, Gesesew HA. Health Care Seeking Behavior in Southwest Ethiopia. PLoS ONE. 2016;11: e0161014. doi:10.1371/journal.pone.0161014

75. Abdu M, Balchut A, Girma E, Mebratu W. Patient Delay in Initiating Tuberculosis Treatment and Associated Factors in Oromia Special Zone , Amhara Region. Pulmonary Medicine. 2020;2020. doi:10.1155/2020/6726798

Fig 4. Map of Jimma Zone, Southwest Ethiopia

Fig 5. Schematic presentation of sampling procedure for a cohort of TB patients, Jimma Zone, 2017/18

Fig 6. Medical provider first visited for the symptoms with patient delay status at Jimma Zone, 2017/18

Supporting information

S1 Text**:** Questionnaire and information sheet with consent form English and local language versions

S2 Text**:** STROB Checklist

Fig 1

Click here to access/download;Figure;Fig 1.tif

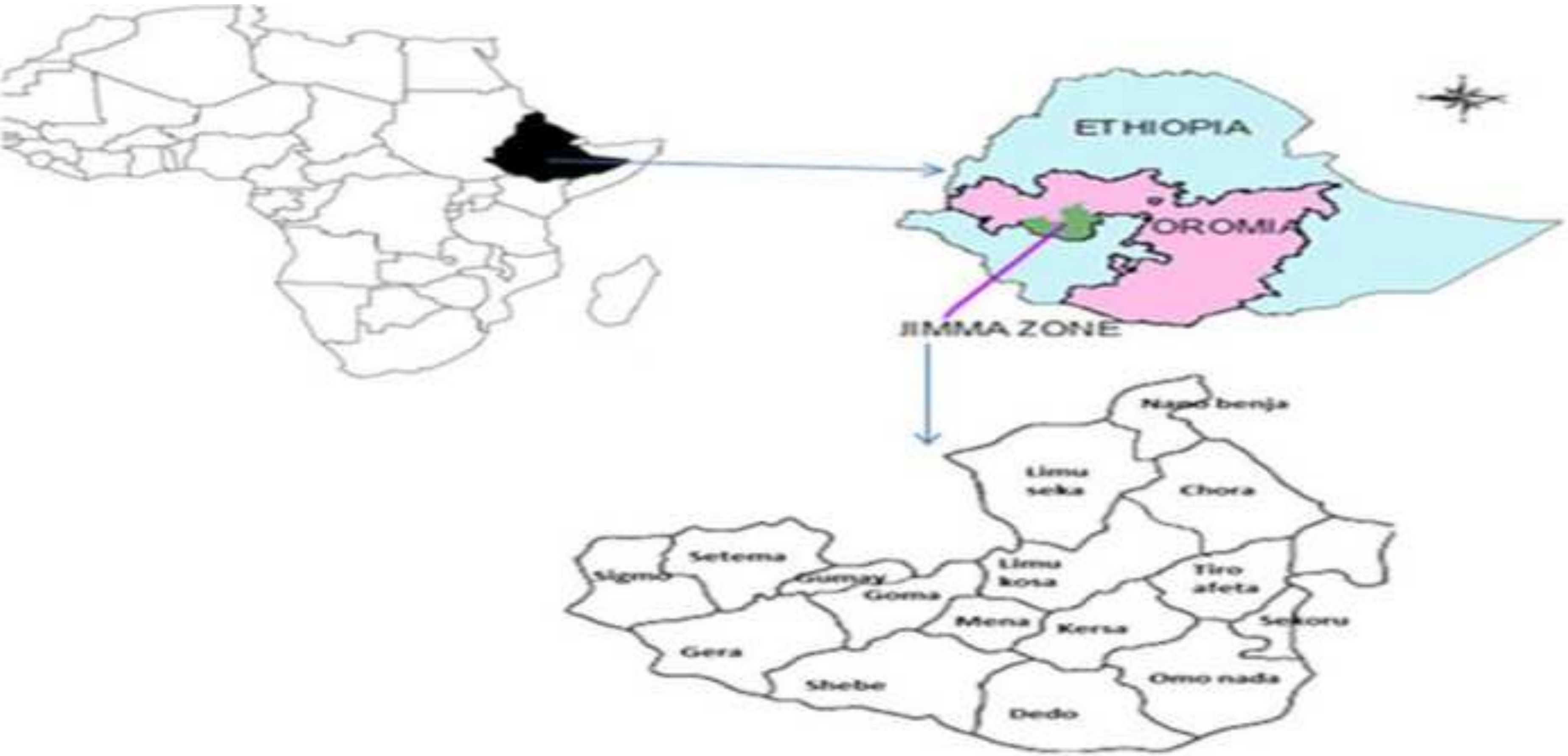

Fig 2

Click here to access/download;Figure;Fig 2.tif

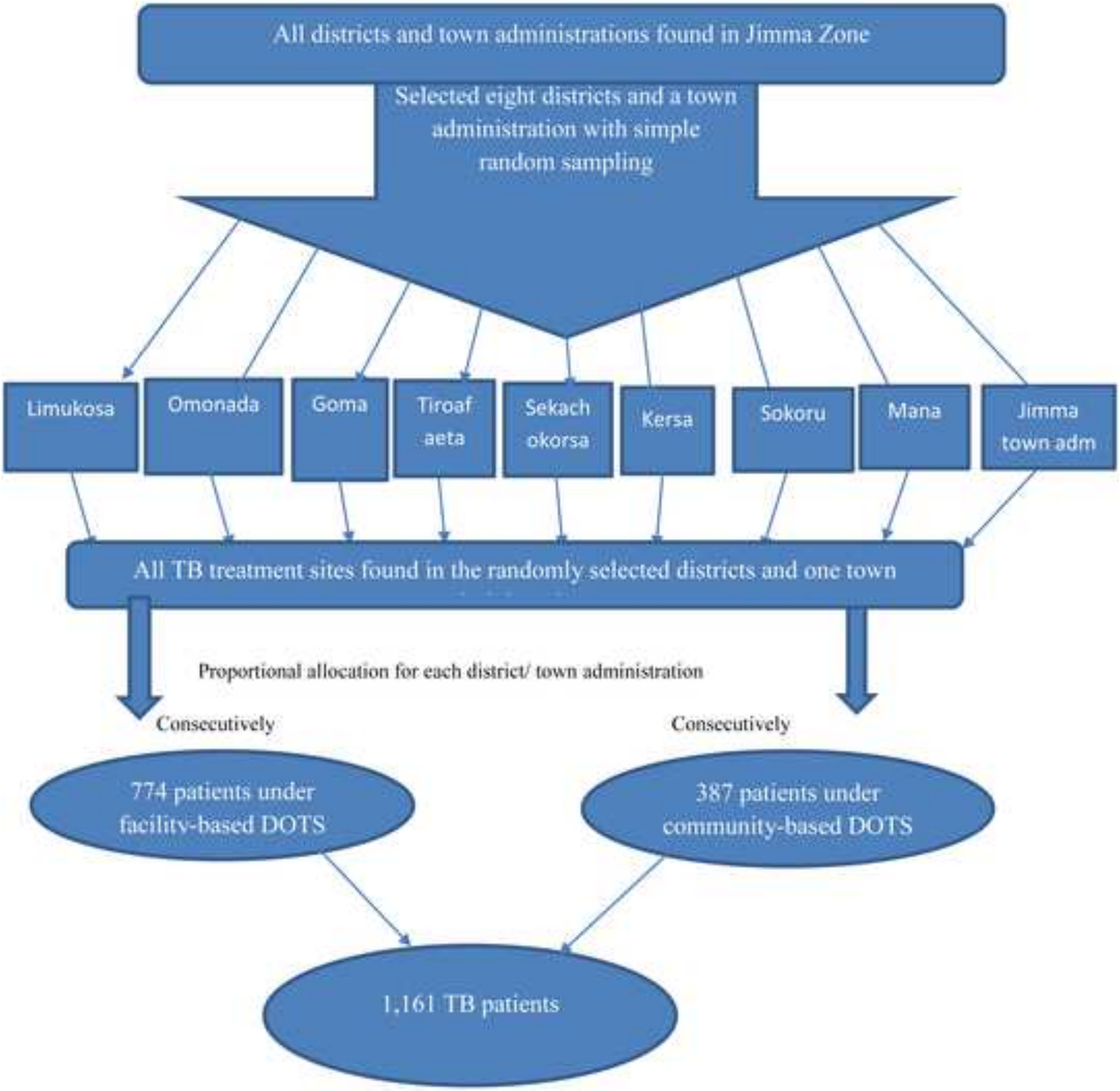

Fig 3

Click here to access/download;Figure;Fig 3.tif

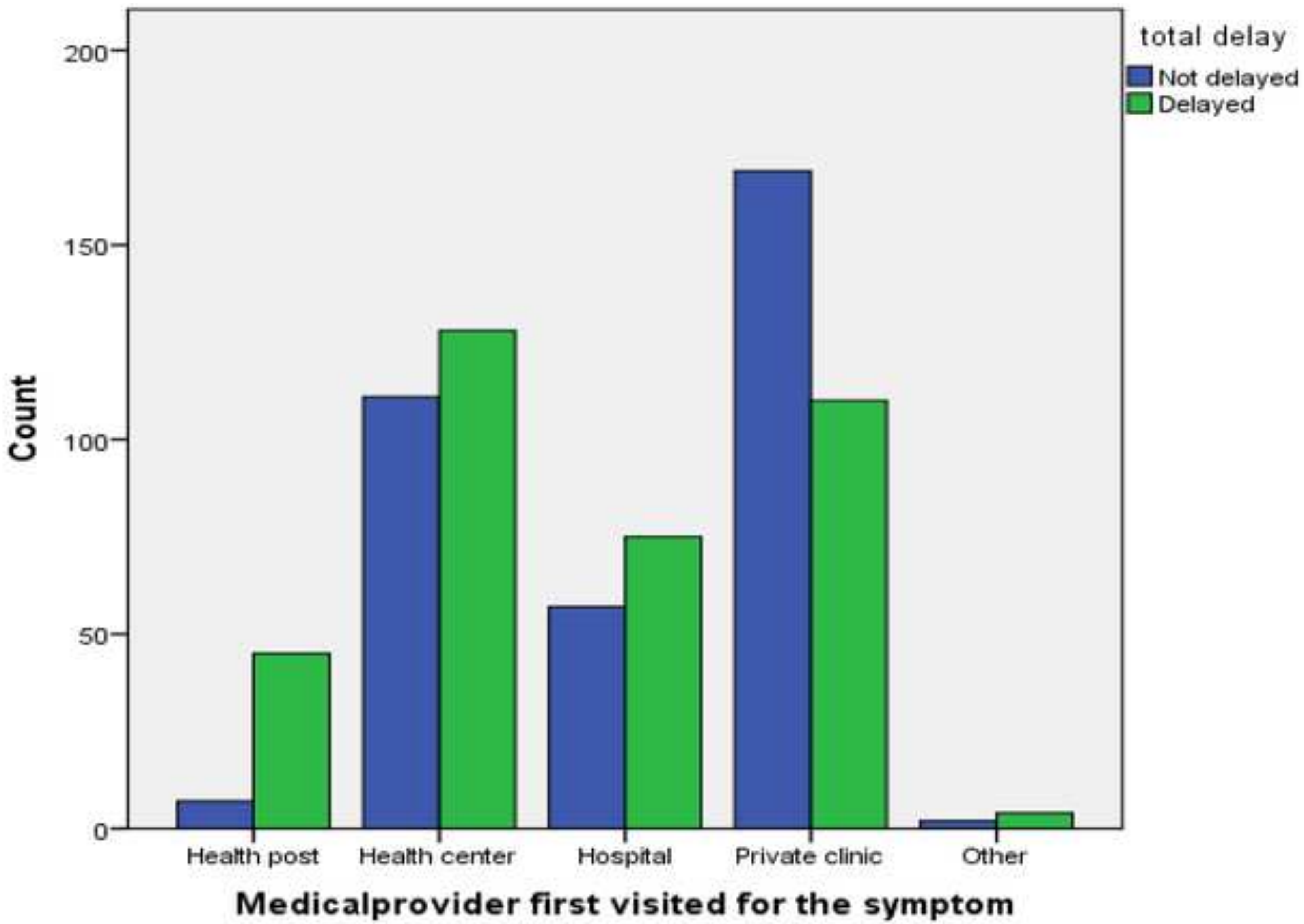

S1 Text

Click here to access/download
Supporting Information
S1 Text.pdf

S2 Text

Click here to access/download
Supporting Information
S2 Text.pdf
