# Supplementary material for: Total delay and associated factors among tuberculosis patients in Jimma Zone, Southwest Ethiopia
Source: PLoS One. 2023 Feb 9;18(2):e0281546. doi: 10.1371/journal.pone.0281546 (PMC10045582; doi:10.1371/journal.pone.0281546)
Supplement: Attachment — Submitted filename: Review.docx [file pone.0281546.s005.docx]

**Authors have performed on TB research by focusing on delay; I have the following points that would be considered**

**Title**

Why authors are interested to total delay… as it did not indicate which delay was important in this research… that is delay related to patients or delay related to health care system?

**In abstract and end of backgrounds**… the aim of this study is not clearly indicated, these also reflected in results and discussion parts … that with open discussion… It would be better if the aim was clear and others were aligned to it.

**In methods**

In line 152, total defined as “the period between onset of TB symptoms and the first start of anti-TB treatment”, in this statement onset of TB symptoms... did it mean five TB symptoms onset (Cough, fever, weight loss, night sweating and Haemoptysis )

**Results**

In line 153, the reason why the median total delay was taken for the classification of delayed or not was not clear

In Table 1… there was “Town”… but in 318… authors used “Urban”… which one is correct? Can we use the terms interchangeable?

From 113 to 114 and in Table 1…authors used the term community based and facility based DOTS, but these terms were not defined

In Table 3, the multivariable analysis for the determinants of delay was indicated, in the Table many variables were indicated? Did that all variables or model run at once or any else? It was not clear indicated in methods part

**Discussion**

Starting from line of 316, how the authors relate the patients followed facility based DOTS were less delayed when compared with community Based DOTS… but in other study published on BMJ open (<https://bmjopen.bmj.com/content/bmjopen/11/7/e048369.full.pdf>) with the same data… CBD was more effective than FBD?

**Conclusion**

In line 350, authors indicted as unacceptable long total delay …. Better if rephrased as there is no acceptable total delay

From line 350-353… the conclusion has no results background

**Reference**

Despite the literatures are available, it would be better if selective
